# Sculpting conducting nanopore size and shape through de novo protein design

**DOI:** 10.1126/science.adn3796

**Published:** 2024-07-18

**Authors:** Samuel Berhanu, Sagardip Majumder, Thomas Müntener, James Whitehouse, Carolin Berner, Asim K. Bera, Alex Kang, Binyong Liang, Nasir Khan, Banumathi Sankaran, Lukas K. Tamm, David J. Brockwell, Sebastian Hiller, Sheena E. Radford, David Baker, Anastassia A. Vorobieva

**Affiliations:** 1 Department of Biochemistry, The University of Washington, Seattle, WA, USA.; 2 Institute for Protein Design, University of Washington, Seattle, WA, USA.; 3 Biozentrum, University of Basel, Basel, Switzerland.; 4 Astbury Centre for Structural Molecular Biology, School of Molecular and Cellular Biology, Faculty of Biological Sciences, University of Leeds, Leeds LS2 9JT.; 5 Structural Biology Brussel, Vrije Universiteit Brussel, Brussels, Belgium.; 6 VUB-VIB Center for Structural Biology, Brussels, Belgium.; 7 Department of Molecular Physiology and Biological Physics and Center for Membrane and Cell Physiology, University of Virginia, Charlottesville, VA, USA.; 8 Molecular Biophysics and Integrated Bioimaging Division, Lawrence Berkeley National Laboratory, Berkeley, CA, USA.; 9 Howard Hughes Medical Institute, University of Washington, Seattle, WA, USA.; 10 VIB Center for AI and Computational Biology, Belgium.

## Abstract

Transmembrane β-barrels have considerable potential for a broad range of sensing applications. Current engineering approaches for nanopore sensors are limited to naturally occurring channels, which provide suboptimal starting points. By contrast, de novo protein design can in principle create an unlimited number of new nanopores with any desired properties. Here we describe a general approach to designing transmembrane β-barrel pores with different diameters and pore geometries. Nuclear magnetic resonance and crystallographic characterization show that the designs are stably folded with structures resembling those of the design models. The designs have distinct conductances that correlate with their pore diameter, ranging from 110 picosiemens (~0.5 nanometer pore diameter) to 430 picosiemens (~1.1 nanometer pore diameter). Our approach opens the door to the custom design of transmembrane nanopores for sensing and sequencing applications.

Transmembrane β-barrel (TMB) nanopores formed by a circularly closed single β-sheet provide rigid scaffolds for the transport of molecules across cellular (1) and organelle membranes (2, 3, 4). Engineering of naturally occurring nanopores has enabled single-molecule enzymology (5), protein finger-printing (6), the detection of small molecules and biomarkers (7), and the sequencing of biological and synthetic polymers (8). Of particular note is nanopore-based DNA sequencing (9), which has enabled widely accessible large-scale genomics, epigenomics, and microbiological analysis (10). However, despite this success the development of nanopore sensors for robust analysis of molecules beyond DNA sequencing has been challenging. The sensing properties of a nanopore for an analyte of interest can be modulated by introducing mutations into the pore lumen that alter nanopore/analyte interactions (11). However, it remains challenging to identify a channel suitable for each of the many applications of interest as there is only a limited set of engineerable naturally occurring nanopores and these have evolved for functions that are, for the most part, very different than the desired applications. Going beyond nature, a conducting pore based on a β-hairpin peptide has been designed that transports poly-lysine peptides (12). Such self-assembling β-hairpins are however not suitable as a general approach to nanopore design as it is challenging to control the channel size and assemble the pore in lipid membranes. Monomeric eight-stranded TMBs have been designed which stably assemble in detergent and in lipid vesicles; however, they are too small to contain a central conducting channel (13).

Encouraged by the success in designing these narrow TMBs, we reasoned that de novo protein design should provide a general approach to creating robust β-barrel nanopore scaffolds for a next generation of nanopore sensors. A key challenge in designing such structures is that the polar-hydrophobic pattern characteristic of globular protein folds must be inverted: the exterior must be largely nonpolar for membrane insertion and the interior must be largely polar to support a solvated conducting channel. Furthermore, unlike globular proteins, the structure of TMBs must be specified primarily by short-range interactions between residues located on adjacent strands because there is no close-packed core. Finally, the amphipathic β-strands are highly aggregation-prone prior to β-barrel assembly and hence the design must strongly favor intra-rather than interchain interactions during folding. We set out to develop general methods to overcome these challenges and design stable monomeric channels with tunable pore shapes, sizes, and single-channel conductance.

## Computational design

**Fig. 1.**
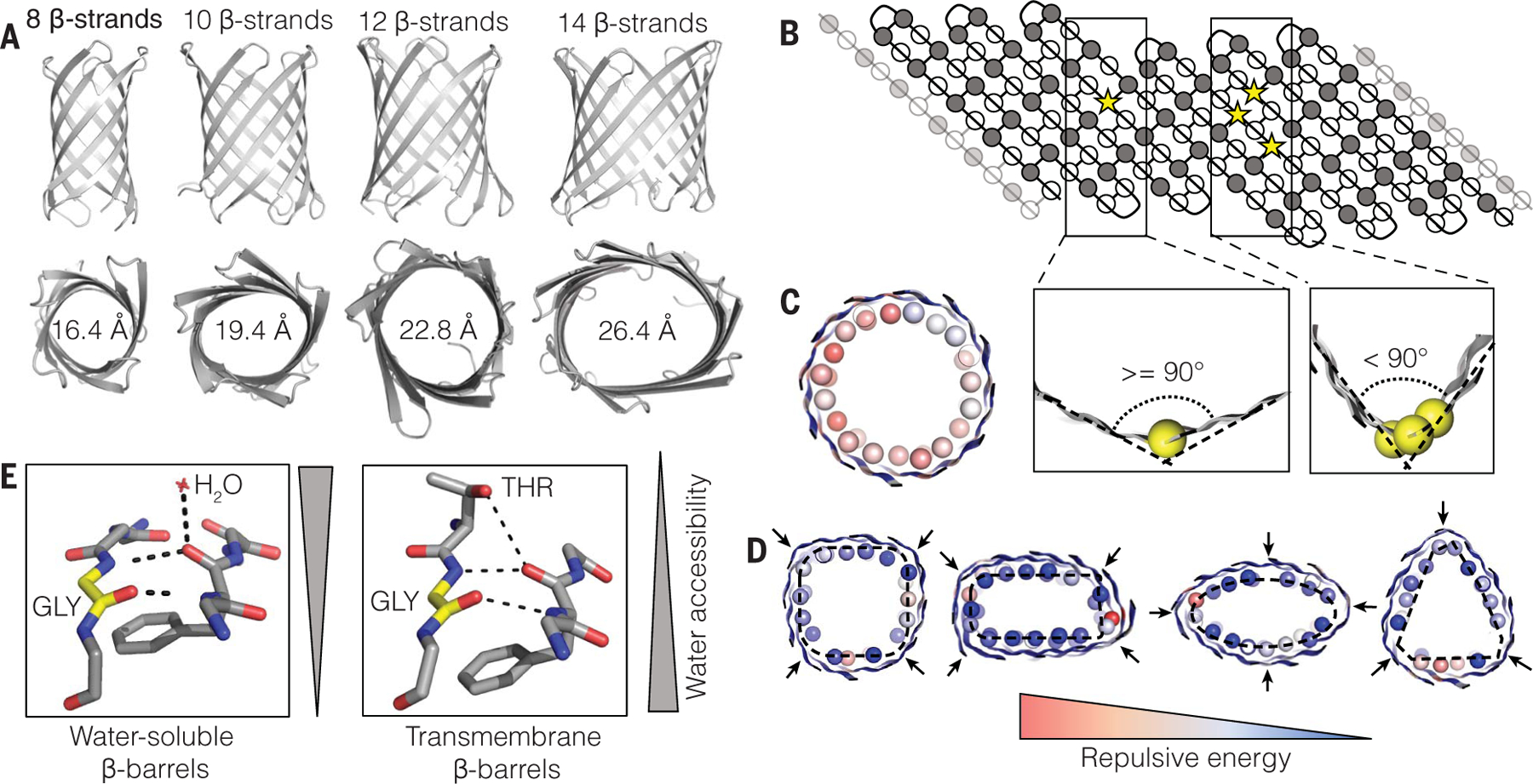
Sculpting β-barrel geometry. (**A**) Barrel diameter can be controlled through the number of β-strands in the β-barrel blueprint. (**B**) β-barrel 2D interaction map. Strong bends in the β-strands (< 90° bend, right) are achieved by stacking several glycine kink residues (yellow spheres) along the β-barrel axis, as opposed to placing one kink (>90° bend, left). (**C** and **D**) Cross sections of explicitly assembled β-barrel backbones without [cylinder in (C)] and with (D) glycine kinks. The Cβ atoms of the residues facing the pore are shown as spheres and colored according to their respective repulsion energy. Glycine kink positions are shown with arrows; placement at the corners of the embedded rectangular, oval, and triangular shapes [dashed lines in (D)] generates the desired backbone geometries. (**E**) Polar threonine residues are tolerated on the membrane-exposed surface of TMBs (right) as they can form a hydrogen bond to the backbone, mimicking the interactions with water molecules observed in similarly curved areas of water-exposed β-strands (left).

We sought to build, from scratch, TMB backbones accommodating water-accessible pores starting from the principles elucidated during the design of eight-stranded TMBs lacking pores (13). To modulate the size of the pore, we increased the number of β-strands (10, 12, and 14 strands) while keeping the transmembrane span and the connectivity between β-strands (the shear number) (14, 15) constant. This resulted in an increase of the average β-barrel diameter from 16.4 Å for the previously designed eight-strand β-barrels (13) to 19.4 Å (10 strands), 22.8 Å (12 strands), and 26.4 Å (14 strands) (Fig. 1A and fig. S1). By comparison to eight-stranded TMBs, the diameters of the larger β-barrels do not allow long-range side chain contacts across the pores and the structural properties of the pores (β-strand pairing, β-barrel shape) must be locally encoded. Naturally occurring TMBs typically feature long, disordered loops on one side of the barrel (16), which can result in noisy electrophysiology recording and challenging data interpretation when the pores are used for sensing applications (17, 18). To design quiet pores and reduce noise we connected the β-strands on both sides of the barrels with 2- and 3-residue β-hairpins (fig. S2), the shortest loops we have previously found to support TMB folding (13). The first-generation backbones corresponding to these designs were assembled with the Rosetta BlueprintBDR (19) application and had similar cylindrical shapes. Such cylindrical β-sheet configurations are strained (20, 21) as a result of repulsion between side chains packing the barrel lumen (Fig. 1C). Glycine kinks (glycine residues in extended positive-φ conformation) (15) were introduced into the blueprint to relieve the strain and to bend the β-strands to form corners in the β-barrel cross section. We generated four blueprints with the same topology but different glycine kink distributions to design 12-stranded β-barrel backbones with square-, triangle-, rectangle-, or oval-shaped cross sections (fig. S1). A single glycine kink was used in corners of an angle of ≥ 90° and several adjacent and/or stacked kinks were placed to form corners of < 90° (Fig. 1B). Sequence-agnostic TMB backbones incorporating these constraints were assembled in silico and had the expected shapes based on the placement of the glycine kinks (Fig. 1D).

A challenge for TMB design is to balance the optimization of the folded β-barrel state in the membrane with delayed folding in water to reduce misfolding and aggregation that would prevent successful integration into a membrane bilayer (13, 22, 23). For the eight-stranded TMBs, this was achieved by incorporating local secondary-structure frustration (24) to reduce premature formation of aggregation-prone β-strands prior to full barrel assembly: hydrophobic amino acids were designed into the water-accessible pore to disrupt the hydrophobic-polar amino acid alternation pattern characteristic of amphipathic β-sheets. To test whether such balancing is necessary for larger β-barrel designs that need to have water-accessible (and hence more polar) channels, we first designed “optimal” 10- and 12-strand TMBs with only polar and charged amino acids facing the pore (table S3). All 16 such designs failed to express in *Escherichia coli* (fig. S6) possibly because they assembled into toxic β-sheet aggregates instead of inclusion bodies, as was previously observed for similarly optimal eight-stranded TMB designs. We therefore set out to design larger TMB nanopores incorporating local secondary structure frustration. In the water-accessible pore, networks of polar residues were designed around the canonical TMB folding motif Tyr-Gly-Asp/Glu (13, 25, 26) to optimize strong local β-register–defining interactions while alternating with patches of hydrophobic and small, disorder-promoting residues (Gly, Ala, Ser; see methods). On the lipid-exposed surface, design calculations favored Ser and Thr in close proximity to a glycine kink where they could form a hydrogen bond to the β-strand backbone, effectively mimicking the backbone-water hydrogen bonds observed in strongly bent β-strands of water-soluble β-barrels (Fig. 1E). Although it is perhaps counterintuitive to expose hydroxyl groups to the lipid environment, we included a small number of these amino acids on the lipid-exposed surface instead of hydrophobic β-branched residues (see methods) to further reduce the β-sheet propensity.

During combinatorial design of sequences for β-barrels of different size we found that the frequency of incorporation of each amino acid type strongly depended on the curvature of the β-sheet. For each of the generated blueprints, we adjusted the Rosetta solvation and reference energies (27) (see methods) to achieve the desired balance of frustrated and energetically favorable contacts (fig. S3). Following several iterations of combinatorial sequence design and structure relaxation, designs were selected based on hydrogen bond network descriptors, secondary structure (28), and aggregation propensities (29) (fig. S4). We previously found that AlphaFold2 with multiple recycles (30) could accurately predict the structures of designed TMBs from single-sequence input without sequence alignments (31) and that the confidence assigned to the model (pLDDT) was a good discriminator of the sequences with higher probability of experimentally folding (32). We selected 4 to 10 designs per blueprint for which AlphaFold2 predicted high-confidence structures closely matching the design models (fig. S5).

## Experimental characterization of TMB folding

**Fig. 2.**
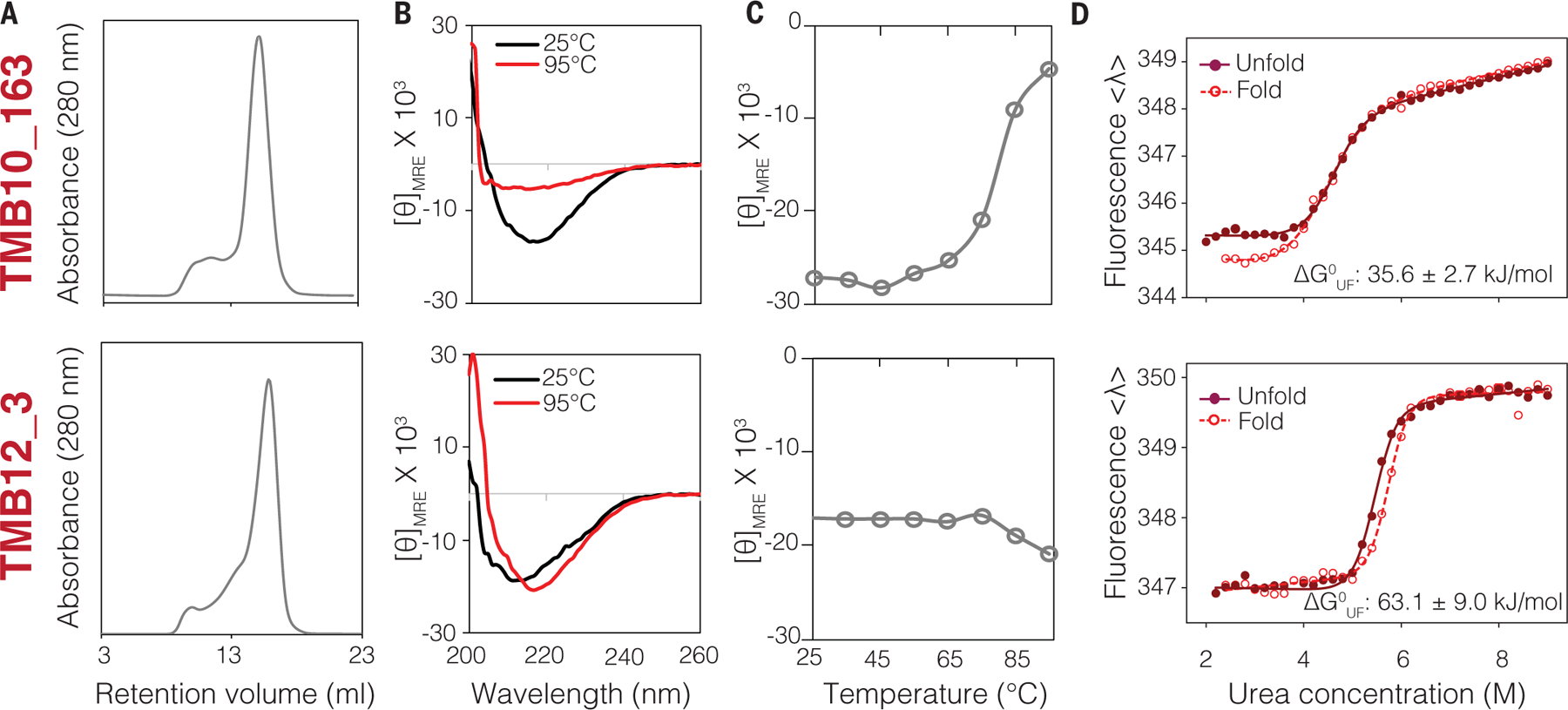
Biophysical characterization of designed nanopores. Top row: 10-stranded design (TMB10_163). Bottom row: 12-stranded design with a square cross section (TMB12_3). Both designs elute as one major species with retention time consistent with a monomeric protein in complex with DPC detergent (**A**) and show distinct negative maxima in far UV CD spectra at 215 nm (**B**) that remain stable up to >70°C (**C**), and cooperative and reversible folding/unfolding transitions in DUPC LUVs [where <λ> is the average tryptophan fluorescence emission wavelength in nanometers (see methods)] (**D**).

We first tested two sets of TMBs with 10 (four designs) or 12 β-strands with a square cross section (nine designs). Genes were synthesized and the proteins were expressed as inclusion bodies in *E. coli* to avoid the complexity of targeting the outer membrane (33) (Fig. 2A). Unlike the 16 “optimal” designs which all failed to express, most sequences incorporating secondary structure frustration were expressed at high levels (12 out of 13, fig. S7). Because most naturally occurring TMBs can fold in vitro (34), the purified designs were solubilized in guanidine hydrochloride and refolded by slow dilution into a buffer containing either detergent [fos-choline 12 (DPC) at a concentration double the critical micellar concentration] or synthetic lipid vesicles (see materials and methods). As previously observed for the eight-stranded TMB designs, the standard band-shift assay on cold SDS-PAGE used to assess folding of natural TMBs (35) was not informative to identify properly folded synthetic TMBs (fig. S8). Instead, the designs were characterized by size exclusion chromatography (SEC), far UV circular dichroism (CD) in the presence of DPC detergent, and tryptophan fluorescence in DUPC (C_11:0_PC) large unilamellar vesicles (LUVs). One 10-strand design (TMB10_163) and one 12-strand design (TMB12_3) with predominantly monomeric SEC profiles (Fig. 2A), thermostable CD spectra characteristic of β-sheet (Fig. 2, B and C) and clear shift of tryptophan fluorescence maximum from ~350 nm (unfolded proteins in 8 M urea or in the absence of lipid) to ~330 nm (folded in LUVs) (figs. S9 and S10) were selected for further characterization by urea titration. Both designs showed sharp and reversible folding/unfolding transitions in the presence of DUPC LUVs (Fig. 2D) [midpoint urea concentrations for folding (Cm^F^): 4.5 ± 0.2 M and 5.5 ± 0.2 M, respectively]. The equilibrium unfolding curves were fitted to a two-state transition, with the calculated unfolding free energies (ΔG^0^_UF_) of −35.6 ± 2.7 and −63.1 ± 8.0 kJ/mol (for TMB10_163 and TMB12_3, respectively) in the range of natural (ΔG^0^_UF_−10 to −140 kJ/mol) (36–39) and previously designed eight-stranded TMBs (−38 and −56 kJ/mol) (13). To confirm that the designs folded by integration into the bilayer rather than partial folding on its surface, the kinetics of folding were recorded in DUPC (C_11:0_PC) membranes as well as in thicker DMPC (C_14:0_PC) membranes. Integral folding is expected to happen more slowly in thicker versus thinner membranes whereas folding on the bilayer surface should be relatively insensitive to its thickness. Substantially decreased folding rates were observed with DMPC compared with DUPC LUVs (fig. S11), consistent with integral membrane folding.

Encouraged by these results, we assessed the nanopore activity of these two designs following spontaneous insertion into planar dipalmitoylphosphatidylcholine (DPhPC) membranes after dilution out of DPC micelles. The 12-strand TMB12_3 was inserted successfully into the membrane and produced distinct jumps of current of reproducible intensities (fig. S12) and stable conductance. Although the design TMB10_163 did not have detectable nanopore activity, the variant TMB10_165 [obtained by sampling surface residues with Rosetta (40) and a modified energy function; see methods] with seven mutations on the lipid-exposed surface (T72V, T102V, I114V, L124A, V126I, V138I, and V144I) inserted into DPhPC membranes and conducted ions (fig. S12). TMB10_165 had higher stability to protease digestion than TMB10_163 and more dispersed nuclear magnetic resonance (NMR) ^1^H-^15^N HSQC chemical shift in DPC micelles (fig. S13). The TMB10_ 165 and TMB12_3 pores remained stably inserted over long periods of time with the longest recording being 2 hours for the TMB12_3 design. Recording of the current-to-voltage response showed monotonic increases in observed conductance with increasing positive or negative voltage, indicative of stable transmembrane channels (I/V curves in fig. S12). Overall, results on TMB10_163, TMB10_165, TMB12_3, and other TMB12 designs with less or no detectable nanopore activity (fig. S15) indicate a strong correlation between membrane integration and nanopore conductance with stable TMB folding in vitro.

**Fig. 3.**
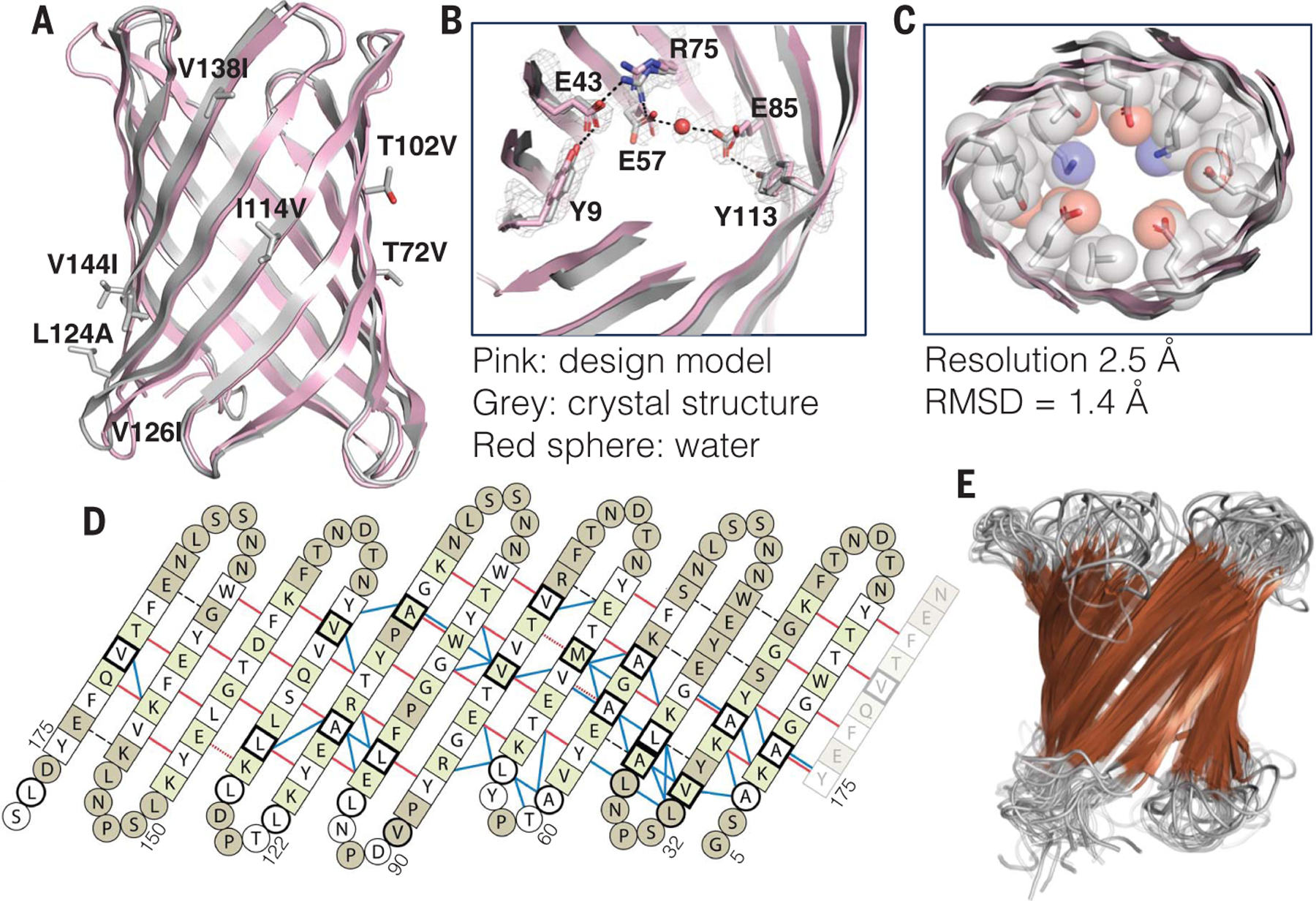
Experimentally determined nanopore structures closely align with the computational design models. (**A** to **C**) Crystal structure of TMB10_163. (A) Backbone superposition. The seven surface residues mutated in TMB10_165 are shown as sticks with the substitution label. (B) Superposition of side chains involved in key folding motifs in the lumen, including *2F*_*o*_ to *F*_*c*_, omit electron density contoured at 1.0 *s*. A water molecule crystallized in the pore is shown as a red sphere. (C) Cross section superposition with residues shown as spheres to highlight the water-accessible pore. (**D** and **E**) TMB2_13 structure in LDAO micelles. (D) Long-range NMR NOE contacts mapped to the expected TMB12_3 hydrogen bonds (dashed black lines). Residues with amide assignment are shown in white and green, unassigned residues are shown in ash gray. Residues with β-sheet secondary structure are shown as squares, all others as circles. Bold outlines indicate available methyl assignments. NOE contacts are shown as red lines (long-range amide-amide, dashes indicate diagonal overlap) and blue lines (contacts involving side chain methyl groups). (E) Ensemble of the 20 lowest-energy solution NMR structures (β-sheets shown in brown).

We next sought to solve the structures of the designs to assess the accuracy of the computational design methods. Although the design TMB10_165 did not form crystals in the conditions screened, TMB10_163 formed crystals which diffracted to 2.5-Å resolution (table S1). The seven surface-exposed mutations between TMB10_165 and TMB10_163 are shown in Fig. 3A. The four copies of the TMB10_163 in the asymmetric unit had a structure similar to the original Rosetta design, with an average RMSD of 1.4 Å over all backbone heavy atoms (Fig. 3A) and featured the expected β-strand connectivity (shear number of 12). Most of the side chains lining the pore had similar rotameric states in the crystal structure and the design model, with notable similarity at the level of the designed Tyr-Gly-Asp/Glu folding motifs (Fig. 3B). Although TMB10_163 nanopore activity was not observed, analysis of its structure using PoreWalker (41) and MOLE 2.5 (42) indicated the presence of a water-accessible cylindrical pore with an average diameter ranging from 4.2 to 5.3 Å in the four subunits (Fig. 3C and fig. S16), matching the diameter of the pore in the TMB10_163 design model (4.6 Å).

We determined the structure of TMB12_3 by NMR spectroscopy. Optimization of the in vitro folding conditions showed that the protein was structured in aqueous solution in LDAO detergent micelles, as indicated by well-dispersed amide and side chain methyl spectra (figs. S17 and S18). Secondary chemical shifts indicated the presence of 12 β-strands as in the design (fig. S19). Amide and side chain methyl NOEs spanned a dense network of experimental connectivities that reached around the barrel circumference and thus confirmed the correct arrangement of the strands into the predicted barrel structure (Fig. 3D). TMB12_3 had the designed β-strands connectivity (shear number of 14) with the barrel closed by the canonical antiparallel β1-β12 seam (Fig. 3E, fig. S20, and table S2).

The crystal and NMR structures demonstrate that our computational design method can design TMB nanopores with precisely controlled shear, channel width, and shape.

## Electrophysiology

Encouraged by our success in designing 10- and 12-stranded β-barrels, we set out to design TMBs with different numbers of β-strands and different shapes. We designed 12-stranded β-barrels with a triangular cross section (eight designs), an oval cross section (seven designs), or a rectangular cross section (nine designs), as well as 14 β-stranded β-barrels (nine designs), incorporating the design features described above for the 10- and 12-stranded TMBs. The designs were obtained as synthetic genes and the proteins were again expressed in inclusion bodies. A lower fraction of 12-stranded TMB designs with a rectangular (4 of 9 designs) and oval (4 of 7 designs) cross section showed a prominent expression band SDS-PAGE gel by comparison to the square-shaped designs (8 of 9). This difference could be the result of a less homogeneous distribution of β-sheet destabilizing amino acids (which are easier to introduce in bent than in flat β-sheet regions) in these designs, as suggested by a higher density of strong β-sheet islands colocalizing with predicted early folding regions (43) (fig. S21). The difficulty of de novo β-barrel design thus depends not only on the size of the TMB pore but also on the shape encoded into the blueprint. We then confirmed that the designs formed soluble, monodispersed species in DPC micelles with expected β-sheet secondary structure (fig. S22) and proceeded to screen them for nanopore activity.

**Fig. 4.**
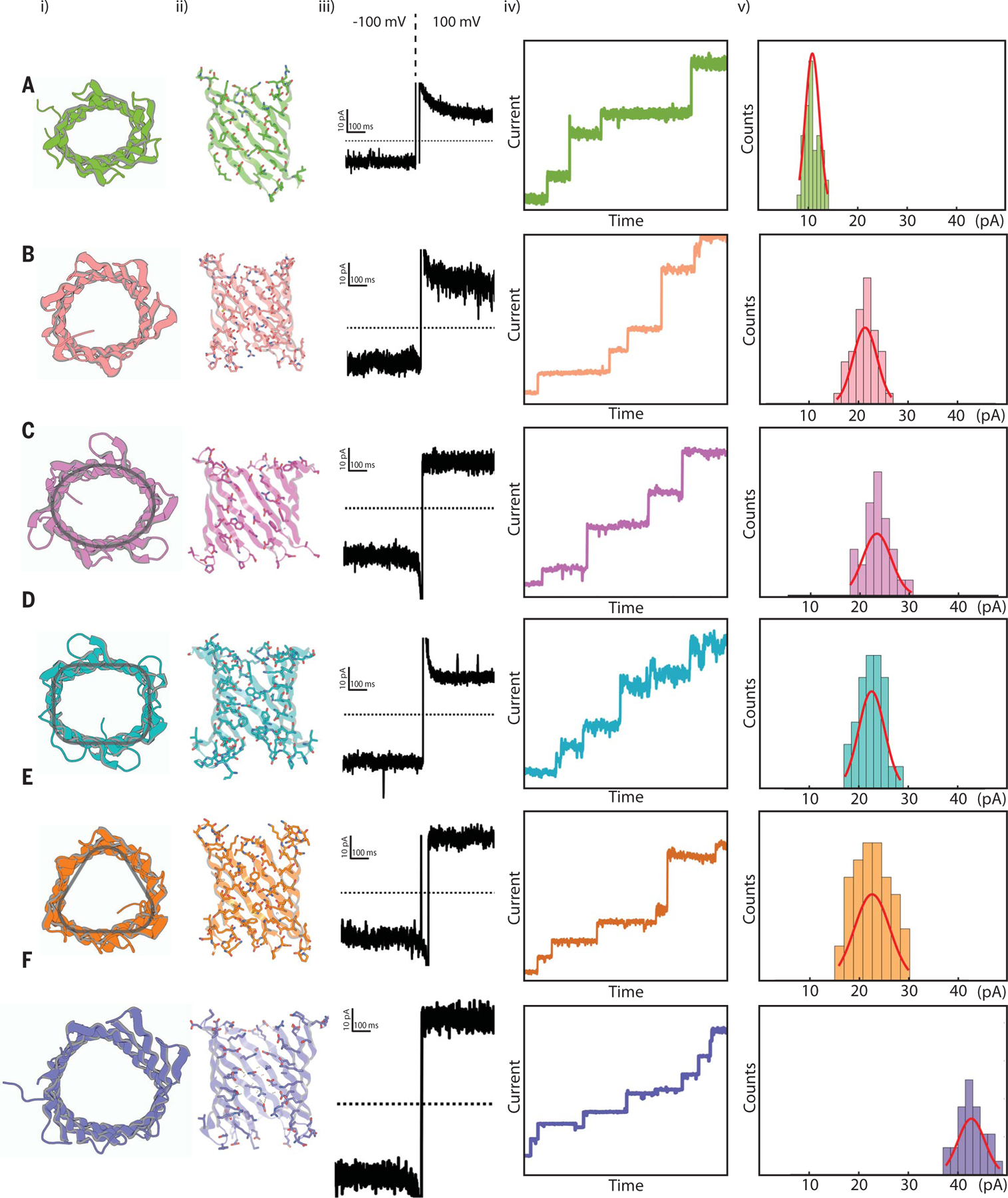
Conductance of designed nanopores. Designs: (**A**) TMB10_165, (**B**) TMB12_3, (**C**) TMB12_oval_4, (**D**) TMB12_rect_8, (**E**) TMB12_tri_12, (**F**) TMB14_8. (i) Top view representation. (ii) Vertical cross sections of the pore. (iii) single channel conductance (smallest observed conductance jump). (iv) sequential insertions of designed pore in planar lipid bilayer membrane from detergent solubilized sample at low concentrations. (v) histogram of smallest measured current jumps for each design, up to 50 pA. The applied voltage across the bilayer was 100 mV and experiments were performed in a buffer containing 500 mM NaCl. A Gaussian fit was carried out for the single channel current histograms for each design. For TMB10_165, 38 independent single channel jumps were identified from three recordings to plot the histogram shown. Similarly, 44 single channel insertions were identified for TMB12_3 (four recordings), 29 insertions for TMB12_oval_4 (three recordings), 30 insertions for TMB12_rect_8 (three recordings), 45 insertions for TMB12_tri_12 (five recordings), and 32 insertions for TMB14_8 (three recordings) to plot the above depicted histograms.

We evaluated the ability of the designs to insert into planar membranes from diluted detergent solution and form conducting pores (Fig. 4). We obtained both 12 (three triangular, three oval, and two rectanglar) and 14 stranded (two) TMBs that exhibited consistent and stable conductances at positive and negative voltage (Fig. 4, 3rd and 5th columns), with multiple sequential insertions corresponding to current jumps of small integral multiples of the base pore conductance (Fig. 4, 4th column).

Based on the intensities of the current jumps, we estimated the conductances of single-channel events, which increase with pore size as expected: the 10-stranded TMB design described above had a conductance of 108 ± 1.4 pS, which based on the cylindrical pore access resistance model (44) corresponds to a nanopore diameter of ~3.5 Å. The 12-stranded designs had similar conductances to each other (210 to 230 pS) despite their different shapes, consistent with a cylindrical nanopore of around 5 Å. The 14-stranded design had a conductance of 427 ± 2.7 pS, consistent with a calculated pore diameter of 7 Å. The predicted diameters are close to the average expected diameters of 4.6 ± 0.7 Å, 9.4 ± 0.8 Å and 10.6 ± 1.4 Å [calculated along the pores of TMB10_165, TMB12_3, and TMB14_8 design models, respectively, using MOLE 2.5 (42) (fig. S16)].

In comparison to naturally occurring pores used for sensing, such as OmpG which undergoes both transient and complete occlusion events by its solvent-exposed loops over a timescale of 100 ms (18, 45), our TMB designs show quiet conductances with no occlusion events detected over 10 s measurements (fig. S12). Varying the shape of the pore while keeping the size constant (Fig. 4, first column) did not have a large effect on monovalent ion conductance, and the net flux of ions is likely more dependent on pore area than shape, given the flexibility of the long polar side chains lining the channel (fig. S23). We anticipate that modulation of the nanopore shape and chemical lining should allow control over the permeability of the pores to larger and more complex solutes in the future.

## Discussion

Our results demonstrate that it is possible to systematically design transmembrane β-barrels with conducting pores spanning a range of sizes and shapes. Despite the inversion of the hydrophobic exterior and polar core compared with globular proteins and the almost entirely local nature of the side chain interactions, our approach enables TMB design with atomic level precision, as highlighted by the close agreement between the experimentally determined crystal and NMR structures and the corresponding design models. Whereas the shapes of globular proteins are largely determined by the packing of hydrophobic residues in a central core, the TMB shapes can be specified by strategic placement of glycine residues at which bending takes place to reduce strain. As previously observed for eight-stranded TMBs, a delicate balance between the optimization of tertiary structure energy and negative design (introduction of locally frustrated residues) to disfavor premature β-strand formation before membrane insertion was critical for the expression of the larger TMB nanopores in *E. coli* inclusion bodies.

In comparison with previously designed oligomeric protein nanopores—built from self-assembling α-helical (46–49) or β-hairpin peptides (12)—the nanopores presented have the advantage of being built from a single chain, which enables assembly of monodisperse nanopores without alternative oligomeric states and with much greater control over the shape of the transmembrane channel (fig. S24), as well as efficient folding into detergent micelles and lipid membranes. Whereas the β-hairpin based nanopores were soluble only in lipid nanoparticles (12), the monomeric TMB design—similar to the naturally occurring nanopores used for sensing applications—can be solubilized in detergent and spontaneously insert into the DPhPC planar lipid membrane following dilution. The most stable nanopores allowed up to 2 hours of quiet recording, thanks to the use of short loops compatible with TMB folding to connect the β-strands. The design principles presented here provide a solution to the long-standing problem of engineering quiet monomeric pores (17, 18, 45, 50) that has limited the use of monomeric integral TMBs such as OmpG as sensors by fusing analyte-recognition motifs (51, 52) or biotin-bound (53, 54) antibodies in the solvent-exposed loops (7). As illustrated in the accompanying manuscript (55), the designed nanopores can be converted into ligand-gated channels with considerably lower noise and more comprehensible signal analysis than previously engineered channels.

Unlike native pores, which are finite in number, there is no limit on the number of distinct designed pores that can be generated. With further optimization of synthetic TMB nanopore insertion into membranes in multichannel flow cells (e.g., by coupling the height of designed nanopores with that of matched thick synthetic membranes) (56), it should be possible to establish fast design-build-test loops to probe the relationship between the chemical properties of a nanopore and the detection of an analyte in the pore lumen (11, 57, 58). Our approach now enables the custom design of pore geometry and chemistry for applications ranging from detection and selective transport of a wide range of molecules of interest to biopolymer sequencing.

## Supplementary Material

SI

MDAR

## Data Availability

The scripts and the designed protein models are available from GitHub (https://github.com/vorobieva/demo_TMB_design) and are archived in Zenodo (59). Analysis scripts for processing ion conductance data as presented in this manuscript are also available on Github (https://github.com/sagardipm/denovoPores) and archived in Zenodo (60).The crystal structure of the design TMB10_163 and the NMR structure of TMB12_3 have been deposited in the Protein Data Bank (PDB) (9FDG, 8UZL) along with corresponding structure factors and NMR restraint data.
