# Supplementary material for: Sculpting conducting nanopore size and shape through de novo protein design
Source: Science. Author manuscript; Available in PMC 2025 Jul 19. (PMC11549965; doi:10.1126/science.adn3796)
Supplement: SI [file NIHMS2028031-supplement-SI.pdf]

Science AAAS

Supplementary Materials for

Sculpting conducting nanopore size and shape through de novo protein design

Samuel Berhanu *et al*.

Corresponding authors: David Baker, dabaker@uw.edu; Anastassia A. Vorobieva, anastassia.vorobieva@vib.be

Science **385**, 282 (2024)
DOI: 10.1126/science.adn3796

The PDF file includes:

Materials and Methods
Figs. S1 to S24
Tables S1 to S4
References

Other Supplementary Material for this manuscript includes the following:

MDAR Reproducibility Checklist

Material & Methods

General purification of all designs

All designs were purified from *E. Coli* following a similar protocol as described previously in *(13)*. Custom genes in a pET29b vector containing the kanamycin resistance gene were ordered from IDT and chemically transformed into BL21DE3 cells. All proteins were purified from inclusion body fractions following complete denaturation in 6M GuCl (Guanidine Hydrochloride) buffer. Briefly, inclusion pellets were washed several times with buffers containing 1% w/v of Triton X-100 and Brij-35 alternatively. A typically washing step involved resuspension of the insoluble pellet in the appropriate buffer, brief sonication and subsequent incubation for one hour at room temperature or overnight at 4°C. After solubilisation of the pellet in GuCl, the protein was diluted to 80-100 uM and refolded in a buffer containing 25 mM Tris-Cl at pH 8.0, 150 mM NaCl and 0.1% DPC (Dodecyl-Phosphatidyl-Choline) either using a dropwise dilution method or by spontaneous dilution to achieve a final GuCl concentration of 0.3 M. The diluted buffer was concentrated after overnight incubation with shaking at 4°C and run on a S200 Cytiva superdex 200 column. Fractions at expected volume were concentrated with a 10 kDa cutoff filter and used for subsequent analysis.

Conductance measurement in planar lipid bilayers

All ion-conductance measurements were carried out using the Nanion Orbit 16TC instrument (https://www.nanion.de/products/orbit-16-tc/) on MECA chips. Lipid stock solutions were freshly made in dodecane at a final concentration of 5mg/mL. DPhPC (Di-Phytanoyl-Phosphatidyl-Choline) lipids were used for all experiments. Designed proteins were diluted in a buffer containing 0.05% DPC (~ 1 CMC), 25 mM Tris-Cl pH 8.0 and 150 mM NaCl to a final concentration of ~100 nM. Subsequently, 0.5 µL or less of this stock was added to the cis chamber of the chip containing 200 µL of buffer while simultaneously making lipid bilayers using the in-built rotating stir-bar setup. All measurements were carried out at 25°C. Spontaneous insertions were recorded over multiple rounds of bilayer formation. All chips were washed with multiple rounds of ethanol and water and completely dried before testing subsequent designs. A 500 mM NaCl buffer was used on both sides of the membrane for all current recordings. Raw signals were recorded at a sampling frequency of 5 kHz. Only current recordings from bilayers whose capacitances were in the range 15-25 pF were used for subsequent analysis. The raw signals at 5 kHz were downsampled to 100 Hz using an 8-pole bessel filter. Estimation of current jumps were carried out using a custom script with appropriate thresholds. Current jumps larger than 2 times the smallest observed jump were discarded for single channel histogram calculations for each design.

Crystallography and structure determination of TMB10_163

SEC purified sample, at the concentration of 14 mg/ml, was used for crystallization. The crystallization screening was performed using a Mosquito LCP by STP Labtech. Crystals grew successfully in 3.25 M 1,6-hexanediol and 0.01 M HEPES pH 7.5. Crystals were harvested directly from a screening tray, and flash cooled in liquid nitrogen. X-ray diffraction was performed at ALS beamline 8.2.1, data were processed with XDS (*61*), and merged/scaled using Pointless/Aimless in the CCP4 program suite (*62*). The structure was phased by molecular replacement using the designed structure as the search model by Phaser (*63*) and refined with Phenix (*64*). Following molecular replacement, the models were improved, and efforts were made to reduce model bias. Structures were refined in Phenix. Model building was performed using COOT (*65*). The final model was evaluated using MolProbity (*66*). Data collection and refinement statistics are recorded in Table S1. Data deposition, atomic coordinates, and structure factors reported in this paper have been deposited in the Protein Data Bank (PDB), http://www.rcsb.org/ with accession code 8UZL.

TMB12_3 Expression for NMR. BL21(DE3) Lemo cells were transformed with a pET29b-derived expression plasmid for TMB12_3. Cells were grown in M9 minimal medium and expression was induced with 0.5 mM IPTG for 22h at 24°C. For expression of [*U*-99% ^{2}H, ^{15}N, ^{13}C]-labeled samples, M9 was prepared with D_2O, $^{15}NH_4Cl$ and deuterated-^{13}C-glucose. For the expression of the [*U*-99%-^{2}H, ^{15}N] labeled sample, M9 was prepared with D_2O and $^{15}NH_4Cl$. For the selectively labeled [^{15}N-Lys] and [^{15}N-Phe] samples, the desired ^{15}N labeled amino acid was added to the culture 45 min before induction.

Purification and Refolding of TMB12_3. Cells were lysed using a M110L from Microfluidics. Inclusion bodies were isolated and dissolved in denaturing buffer (20 mM Tris/HCl pH 8, 150 mM NaCl, 6M GdnHCl), then dialyzed against H_2O in a 10,000 MWCO dialysis membrane for 2h, followed by centrifugation at 30,000 g to precipitate the protein. Precipitated TMB12_3 was dissolved in 10 mM Tris/HCl pH 8, 7 M urea. Refolding was done at 4°C by dropwise rapid dilution into a stirred refolding buffer (20 mM Tris/HCl, 2 mM EDTA, 0.6 M L-Arg, 15 mM LDAO, pH 10). The dilution ratio was set to 1:20 and after overnight stirring, the refolded protein was dialyzed against 20 mM NaPi pH 6.8, 1 mM EDTA for 2h. The refolded protein was concentrated with MWCO 10,000 and the sample was loaded on an S200 size exclusion column, pre-equilibrated with 20 mM NaPi, 1 mM EDTA, 15 mM LDAO. The fractions containing protein were pooled and concentrated using MWCO 10,000.

Isotope labeling samples. The following samples were made: [U-99%-^{15}N]-TMB12_3 in LDAO, [U-99%-^{2}H, ^{15}N, ^{13}C]-TMB12_3 in LDAO, [U-99%-^{2}H,^{15}N; 99%-$^{1}H^{\beta}$,$^{13}C^{\beta}$-A; 99%-$^{1}H^{\varepsilon}$,$^{13}C^{\varepsilon}$-M; 99%-$^{1}H^{\delta1}$,$^{13}C^{\delta1}$-L; 99%-$^{1}H^{\gamma1}$,$^{13}C^{\gamma1}$-V]-TMB12_3 in [U-99%-^{2}H]-LDAO, [^{15}N-Lys]-TMB12_3 in LDAO, [^{15}N-Phe]-TMB12_3 in LDAO. The final sample conditions were 20 mM $Na{\cdot}PO_4$, 1 mM EDTA, pH 6.8, 300–500 mM LDAO, 0.2–1 mM TMB12_3.

NMR experiments. All experiments were carried out at 25°C on Bruker spectrometers operating at field strengths of 700, 800 and 900 MHz. All spectrometers were equipped with a cryogenic triple-resonance probe. The following experiments were recorded: 2D [^{15}N, ^{1}H]-BEST-TROSY (*67*), 3D BEST-TROSY-HNCACB with ^{2}H decoupling (*67*), 3D [^{1}H,^{1}H]-NOESY-^{15}N-TROSY (*68*), 2D ^{13}C-Methyl-SOFAST (*67*), 3D [^{1}H,^{1}H]-NOESY-^{13}C-HMQC (*69*).

Structure calculation. Structure calculation was performed with CYANA 3.98.15 (*70*). All spectra were processed and analyzed with NMRPipe (*71*) and ccpNMR version 3 (*72*). 129 dihedral constraints were derived by TALOS-N (*73*) from the experimentally determined C_{α}, C_{β}, N and HN chemical shifts. Only TALOS-N predictions classified as "strong" were used. Tolerances were set to one standard deviation, capped at a maximum of 20°. A total of 205 experimental NOEs were obtained from 3D [^{1}H,^{1}H]-NOESY-^{15}N-TROSY and 3D [^{1}H,^{1}H]-NOESY-^{13}C-HMQC spectra. 97 Hydrogen bond constraints were inferred from the measured NOE data with upper limits of 2.0 / 3.0 Å and lower limits of 1.8 / 2.7 Å for the HN…O and N…O, respectively. In regions with sparse assignment, these constraints were inferred indirectly from the experimentally established β-strand topology (dashed black lines in Figure 3D). A total of 500 structures were calculated and the ensemble of 20 lowest energy structures was selected. Ramachandran statistics of this ensemble showed 81.1 % of residues in most favored regions, 17.6 % in allowed regions, 1.2% in generously allowed regions and 0.1% in disallowed regions.

Calculating the theoretical diameters of nanopores. The diameters of the nanopores were inferred from the observed single-channel conductance (*G* (nS)) using the access resistance model (**eq. 1**), where R is the resistance, S the conductivity of the solution (S/m), d is the diameter of the pore (nm) and L is the pore length (nm). The purely geometric model approximates the

properties of a cylindrical nanopore and assumes homogeneous solution, pore and membrane neutrality and constant potential at the pore mouth.

$$G = \frac{1}{R} = \frac{S\pi d^2}{4L} \qquad \text{eq. 1}$$

A pore length of 3.5 nm was used for all calculations based on the total transmembrane span of TMB designs. The conductivity of a solution of 0.5 M NaCl was estimated to 40.5 mS/cm based on the previously reported relationship between NaCl molarity and conductivity of the solution.

Folding kinetics measured by Tryptophan fluorescence. Protein samples were buffer exchanged into unfolding buffer (50 mM Glycine-NaOH pH 9.5, 8 M/10 M urea) using a 0.5 mL ZebaSpin 7K MWCO desalting column. The concentration was determined by nanodrop. For kinetic experiments, 15 µL of unfolded protein was added to 485 µL of pre-warmed (25°C) pre-fold buffer of LUVs in 50 mM glycine-NaOH, pH 9.5, in a QS quartz cuvette to give final concentrations of 0.4 µM OMP, 600-3200 LPR (*mol:mol*), 0.24-4 M urea, 50 mM glycine-NaOH pH 9.5. Immediately after mixing, a time based fluorescence scan was carried out on a PTI QuantaMaster™ spectrofluorometer (Photon Technology International), controlled by FelixGX v4.3 software, with excitation at 280 nm, and emission measured at 335 nm. The slit settings were 0.5 nm for excitation, and 5 nm for emission, to minimize photobleaching. Integration was set at 1 s between time points, and the temperature was maintained at 25°C throughout. Fluorescence emission spectra were measured by exciting tryptophan at 280 nm, and measuring fluorescence emission between 300-400 nm, using the same slit-width settings as above, with samples in urea concentrations between 0.24-9.9 M urea.

Circular dichroism in liposomes. Protein samples were prepared in a similar manner as for the tryptophan fluorescence samples. Samples were made to a 600:1 (*mol:mol*) LPR, with final concentrations of 4 µM TMB, 1.2 mM lipid-LUV, 0.24-8 M urea, 50 mM glycine-NaOH pH 9.5 in a final reaction volume of 300 µL. The reaction was allowed to proceed overnight at 25°C to maximize the fraction of protein folded into the lipid-LUV bilayer. Controls were made where the volume of substrate was replaced with 50 mM glycine-NaOH pH 9.5 and an appropriate volume of urea to match the protein samples. These were used to normalize the data by subtracting their CD signals from the CD signal from the protein containing samples. Measurements were taken using 300 µL of sample in a 1 mm QS quartz cuvette, using a Chirascan plus CD Spectrometer (Applied Photophysics). The bandwidth was set at 2.5 nm, and used adaptive sampling to adjust the integration time for the optimal signal:noise. Four scans were averaged between 260 nm to the lowest useable wavelength for each respective sample, which was the point where the voltage reached its upper limit of 1000 V, after which the data became unusable. During temperature ramp experiments, only single scans were taken as the temperature ranged between 25°C and 87°C.

Equilibrium denaturation analysis. To determine the urea dependence of TMB folding, urea denatured TMBs in 50 mM glycine-NaOH pH 9.5, 10 M urea were diluted into DUPC LUVs at an Lipid-to-Protein ratio (LPR) of 600:1 (mol/mol) to give a final concentration of 0.4 µM TMB in 50 mM glycine-NaOH pH 9.5 containing 2-9.9 M urea, and folding was allowed to proceed overnight at 25°C. For urea dependence of unfolding, TMBs were folded in DUPC LUVs (LPR 600:1 (mol/mol)) in 50 mM glycine-NaOH pH 9.5, 2 M urea overnight at 25°C. Pre-folded TMBs were then unfolded by dilution into 50 mM glycine-NaOH pH 9.5 containing 2-9 M urea to a final TMB concentration of 0.4 µM and incubated overnight at 25°C. Tryptophan fluorescence emission spectra were obtained using a PTI QuantaMaster spectrofluorometer (Photon Technology International) in QS quartz cuvettes with excitation slits set to 1 nm and emission slits set to 5 nm. Fluorescence was excited at 280 nm and emission spectra were acquired between 300-400 nm using a step size of 1 nm and an integration time 0.5 seconds. Average wavelength between 325-

375 nm was calculated using equation 2, where <λ> is the average wavelength, I_λ is the fluorescence intensity at a given wavelength, λ is the wavelength, and ∑I is the sum of the intensity of the entire emission spectra.

$$< \lambda > = \frac{\Sigma I_\lambda * \lambda}{\Sigma I} \qquad \text{eq. 2}$$

The experimental data were fitted to a 2-state transition model (*74*) to extract ΔG_0 (the Gibb's free energy for unfolding in the absence of denaturant), the m-value (m_{UF}, the global dependence of ΔG_0 on the concentration of denaturant and C_m (the transition midpoint) based on Obs_F and Obs_U (the observed <λ> for the folded and unfolded states in the absence of denaturant ([D]=0)), m_f and m_u (the linear dependence of Obs_F and Obs_U to [D]). The observed <λ> was corrected to account for the difference in quantum yield between the folded and unfolded states based on the Q-factor (QF), calculated by taking the ratio of the summed fluorescence intensities at the folded and unfolded states. R is the universal gas constant and T is the absolute temperature.

***De novo* backbones assembly.** The entire computational design pipeline has been described in detail in (*75*). The Rosetta blueprint representations of the beta-barrel backbones were generated based on user input using a custom python script available on GitHub https://github.com/vorobieva/demo_TMB_design/tree/master/generate_blueprint. The script requires the SciPy and BioPython modules and generates a Rosetta blueprint (describing local secondary structure and torsion angle bins per residue) and a Rosetta constraints file (describing backbone-backbone hydrogen bonds in the barrel). Examples of blueprint and constraints files used in this study are available on GitHub https://github.com/vorobieva/demo_TMB_design/tree/master/12_strands_square/assemble_backbones. The backbones were assembled based on such a blueprint and constraints file using the Rosetta BluePrintBDR application (*19*), alternating between sampling of backbone fragments and minimization with hydrogen bonds constraints. The highest-quality protein backbones (250-500 backbones, based on Rosetta `vdw`, `omega` and `rama_prepro` scores) were selected as template for combinatorial sequence design.

Combinatorial TMB sequence design. *De novo* β-barrel backbones assembled using the Rosetta coarse-grained centroid model were subject to one round of fast atomistic refinement of the backbones with the Rosetta full-atom model (`ref2015` energy function with limited sampling depth `-nstruct 1` and limited sequence space). The Tyr-Gly-Asp/Glu TMB folding motifs were then designed into the structures using the HBNet (*76*) Rosetta application, which finds all possible positions of the hydrogen bond acceptor residue in the motif (Asp or Glu) based on defined tyrosine positions. The refined TMB backbones were used as templates for several rounds of combinatorial sequence design, alternating between the water-accessible pore (two rounds of design) and the lipid-exposed surface (three rounds of designs) using re-fitted energy functions (see below). Rosetta resfiles were used to define the set of amino acids sampled at each position in the protein. The surface-exposed residues were constrained to mostly hydrophobic amino acids while all amino acids but PRO and CYS were allowed at pore-lining positions. The β-turn residues were designed using previously identified canonical TMB β-turn sequences (Figure S2). At each iteration of sequence optimization, the whole population of designs was analyzed, population-wide selection metrics were computed (see below) and around 10 % of the designs were selected to be used as input for the next iteration. After an iteration of pore-residue design, the outputs were selected based on backbone quality metrics (Rosetta `omega`, `rama_prepro`, `hbond_lr_bb` scores) and on the computed `total` and `hbond_sc`

energies of the hydrogen bond acceptor residues in the Tyr-Gly-Asp/Glu folding motifs. After an iteration of surface-exposed residue design, the outputs were selected based on Rosetta `total_energy` and on the retention of the Tyr-Asp/Glu interactions in the designed folding motifs (which are repacked while the surface residues are designed). The resfiles used in this study to generate the different TMB architectures are available on GitHub (https://github.com/vorobieva/demo_TMB_design/tree/master). A complete description of the design pipeline, analysis scripts and example inputs are available on GitHub (https://github.com/vorobieva/demo_TMB_design/tree/master).

Re-fitted TMB-specific energy functions. To fine-tune the amino acid propensities to TMB-specific statistics, the Rosetta reference energy function (`ref2015`) was modified by testing several variations of weights on one representative TMB backbone. To generate an energy function to design the water-accessible pore-lining residues, the weight of the Rosetta full-atom solvation energy (`fasol`), electrostatic energy (`faelec`) and of the reference energies of small disorder-promoting amino acids (ALA and SER) were systematically varied. The scoring function used for subsequent sequence design was selected based on the closest match between the resulting designed sequences and naturally-occurring TMB sequences at the level of the overall hydropathy of the pore and the frequency of ALA and SER amino acids. To generate an energy function to design the lipid-exposed surface residues, the weight of the Rosetta full-atom solvation energy and of the reference energies of large hydrophobic (PHE) and of small disorder-promoting amino acids (GLY and ALA) were systematically varied. A scoring function was selected based on the closest match between the resulting designed sequences and naturally-occurring TMB sequences at the level of the overall hydropathy of the surface and the frequency of ALA and GLY amino acids. The TMB10 variants (TMB10_163-6), differing by only a few surface mutations, were obtained by resampling hydrophobic and Threonine residues on the β-barrel surface using Rosetta FastDesign ('round4' energy function and resfile available in the GitHub repo) and selecting low-energy models.

Comparison of the scoring weights optimized to design the pore residues of TMB12s designs

	TMB12_square	TMB12_oval	TMB12_rectangle	ref2015
fasol	0.9	0.9	0.9	1.0
faelec	1.4	1.45	1.45	1.0
ALA refE	0.4	-0.6	-0.6	1.32468
SER refE	-1.2	-1.8	-1.8	-0.28969

Designs validation and selection. The final designs were filtered based on the desired balance between local secondary structure frustration (β-sheet propensity of the sequence between 30 % and 50 % (predicted with RaptorX (*28*)) and aggregation propensity score predicted with Tango (*29*) smaller than 1500) and sequence-encoded tertiary structure. The sequence/structure compatibility was assessed based on the capacity of AlphaFold2 (*30*) to fold the sequence into the designed TMB structure in single sequence mode (no multiple sequence align input) and using 48 recycles through the network. Selected predictions had AlphaFold2 plDDT scores higher than 0.8 and showed high structure similar to the expected design model when superimposed with TMAlign (*77*), with Root Mean Square Deviations (RMSD) of < 2.1 Å.

Supplementary Table

Table S1. X-ray diffraction data collection and refinement statistics

	TMB10_163 (8UZL)
Resolution range	49.92 - 2.5 (2.58 - 2.5)
Space group	*P* 2_1
Unit cell	55.62, 52.55, 100.05, 90, 93.67, 90
Unique reflections	20045 (1961)
Multiplicity	4.3 (4.5)
Completeness (%)	98.87 (98.54)
Mean I/sigma (I)	4.3 (0.4)
Wilson B-factor	43
R-merge	0.126 (0.923)
R-pim	0.069 (0.493)
$CC_{1/2}$	0.99 (0.83)
Reflections used in refinement	20021 (1960)
R-work	0.227 (0.336)
R-free	0.262 (0.383)
Number of non-hydrogen atoms	4535
macromolecules	4420
ligands	16
solvent	99
Protein residues	568
RMS (bonds)	0.002
RMS (angles)	0.45
Ramachandran favored (%)	96.96
Ramachandran allowed (%)	3.04
Ramachandran outliers (%)	0.00
Average B-factor	52
macromolecules	52
ligands	52
solvent	50

Statistics for the highest-resolution shell are shown in parentheses.

Table S2. NMR and refinement statistics for TMB12_3 in LDAO micelles

	TMB12_3 (9FDG)
NMR distance and dihedral constraints	424
Distance constraints	302
Total NOE	205
Intra-residue	22
Inter-residue	183
Sequential ($\lvert i - j \rvert = 1$)	59
Medium-range ($\lvert i - j \rvert < 4$)	44
Long-range ($\lvert i - j \rvert > 5$)	102
Intermolecular	0
Hydrogen bonds	97
Total dihedral angle restraints	122
f	61
y	61
Structure statistics	
Violations (mean and s.d.)	
Distance constraints (Å)	0.04 +/- 0.01
Dihedral angle constraints (°)	0.9 +/- 0.4
Max. dihedral angle violation (°)	6.3 +/- 3.1
Max. distance constraint violation (Å)	0.71 +/- 0.22
Ramachandran plot statistics	
Residues in most favored regions	81.1 %
Residues in additionally allowed regions	17.6 %
Residues in generously allowed regions	1.2 %
Residues in disallowed regions	0.1 %
Average pairwise r.m.s. deviation* (Å)	
Heavy	2.94 +/- 0.37
Backbone	2.28 +/- 0.41

* Calculated among 20 refined structures

Table S3. "Optimal" designs that failed to express

Designs	Amino acid sequence	BLAST<br>E-value
**TMB10_r1_1**	MSNSPGARLNIIFRYRSDGTFELTIQFGFKFTVYPEVSIEFQFG<br>VTYKGDSIVEFSDTLGFEFKVYPTLSFYFGFGIQYEDSNRFALT<br>FKFGVKFKLWDPIRLDITVGIRYAGDDSVTVYVGVTLEIQVYPS<br>FEVYFGFGVEYIGESILKFGFEIGFTYW	0.022 (unnamed protein [*Nesidiocoris tenuis*])
**TMB10_r1_2**	MDRSPGTRIRIEVTYASDGDFQITVTVGIEFTLYPEITFEIAVG<br>FTYRGDSKFAIRFEIGVKFKVYPTILVYFGFGIDWSESNEFEIV<br>FEIGFRVKVYDPFEIELRFGVKYQGLDVFTIYAGITFHFTVYPS<br>VTFYVGFGFEYFGDSIVRFGVTFGFQYN	0.05 (unnamed protein [*Nesidiocoris tenuis*])
**TMB10_r1_3**	MKPSPGLSIEITVQYNSDGSVDFTVKVGFTIFVYPTVAVTFEVG<br>VTYHGDSRVTFHFTFGFTVTVYPTLTFYAGFGFQWEDSNTFAIQ<br>FDFGFTIKLYDPVRIYFDAGFAYKGDDKVTFYAGFALQITVYPS<br>VEFYVGVGFQYTGDSTSNAGIRFGVQYK	No match
**TMB10_r1_4**	MKPSPGVRIIIEVQYNSDGSVDFTVKVGFTIFVYPTVAVTFEVG<br>VTYRGDSTVTFHFTFGFSVTVYPTLTFYAGFGFTWDDSNRFAIQ<br>FEAGFTITLYDPVQLYFDFGFTYAGDDKVTFYAGFALQVQVYPS<br>VKFYVGVGFEYTGDSKSNAGIKFGVQYD	No match
**TMB10_r1_5**	MKPSPGARIVVEVQYNSDGSVDFRVKVGFTIFFYPTLAVTFEVG<br>VTYRGDSRVTLHFTFGFTVTVYPTLTFYAGFGFTWKDSNTFAIQ<br>FDAGFTIKLYDPIRLYFDFGFQYAGDDKLDFYAGFAIEFDVYPS<br>VTFYVGFGAEYTGDSTSNAGVKFGVTYY	0.044 (unnamed protein [*Closterium sp.*])
**TMB10_r1_6**	MKPSPGVRIIIEVQYNSDGSVDFTVKVGFTIFFYPTVAITFEVG<br>VTYRGDSTVTFHFTFGFSVTVYPTLTFYAGFGFTWDDSNRFAIQ<br>FEAGFTITLYDPVQLYFDFGFTYAGDDKVTFYAGFALQIQVYPS<br>FKFYVGVGFEYTGDSKSNAGIKFGVQYD	No match
**TMB10_r1_7**	MKPSPGVRIIVEVQYNSDGSVDFTVKVGFTIFFYPTVAVTFEVG<br>VTYRGDSTVTFHFTFGFSVTVYPTLTFYAGFGFTWDDSNRFAIQ<br>FEAGFTITLWDPVQIYFDFGFTYAGDDKVTFYAGFALQIQVYPS<br>FKFYVGVGFEYTGDSKSNAGIKFGVQYD	No match
**TMB10_r1_8**	MKPSPGVRIIIEFQYNSDGSVDLTIKVGFTIFVYPTVAVTFEVG<br>VTYRGDSTVTFHFTFGFSVTVYPTLTFYAGFGFTWDDSNRFAIQ<br>FEAGFTITLYDPVQIYFDFGFTYAGDDKVTFYAGFALQIQVYPS<br>VKFYVGVGFEYTGDSKSNAGVKFGVQYD	No match
**TMB12_r1_11**	MSQSPGLKFYITFRWNSDGKLQVEVGFKIELFFYPTVFISLIFG<br>FVWDGDSKLFFQISDGVEFQVWPDLSFYFGAGIRWSSSNKIEIE<br>FKAGFKIRLYDPFEVIFTFGFTWKGDSTLAIQFGITFTFTFYPT<br>FKIQFGFGVQWEGDSKVEFTFGFEIEVKVYPPVKFRFGAGVIWV<br>GDSQLHVGVYVGYEIK	0.038<br>(unnamed protein [*Nesidiocoris tenuis*])

**TMB12_r1_12**	MDPSPGVRFYVEFIWRSDGEVTFKAGIQFIFIVYPELTITFEIG FTWEGDSTFAIRVTFGFKVRFWPTLEVYVGFGVQWAEDNKIRFE FKAGIIIKIYDPITLEFRFGVTWFGDSIVKIDFGVAITFRVYPT FEITFGVGATWQGDSKFAINIGVEFRFTLYPPAIITVGFGFTWE GDSTLKFGFYFGYTIE	0.046 (unnamed protein [*Nesidiocoris tenuis*])
**TMB12_r1_13**	MQDSPGLNFYIKIEWRSDGFLLIFVGFRIQIFLYPNFTIEIEIG VVWQGDSLFSIRITFGFIINLWPTVQFYAGVGVEWSSSNKFTIT FEAGFTIKFYDPLRIVFFFGFQWEGDSKLTIRAGFTVQITFYPS FQVEFGFGFVWFGDSILVFRFGFRIEVQVYPPATVEFGVGVDWR GDSIVTVGVYFGYKIH	0.04 unnamed protein [*Closterium sp.*])
**TMB12_r1_14**	MDPSPGVRFYVEFIWRSDGEVTFKFGIQFIFIVYPELTITFEIG ATWEGDSTFAIRVTFGFKVRFWPTFEVYVGFGVQWAEDNKIRFE FKAGIIVKLYDPITLEFRFGVTWFGDSIVKIDFGVAITFRVYPT FEITFGVGATWQGDSKFAINIGVEFRFTFYPPAIFTFGFGFTWE GDSTLKFGFYFGYTIE	0.048 unnamed protein [*Nesidiocoris tenuis*])
**TMB12_r1_15**	MDPSPGVRFYFEVIWRSDGEVTIKFGIQFIFIVYPEFTLTFELG ATWEGDSTFAIRVTFGFKIRIWPTVEFYFGFGLQWAEDNKLRFE FKAGIIIKIYDPITLEFRFGLTWFGDSILKVDFGVAITFRVYPT FEITFGVGATWQGDSKFAINIGVEFRFTVYPPAIITVGFGFTWE GDSTLKFGFYFGYTIE	0.025 unnamed protein [*Nesidiocoris tenuis*])
**TMB12_r1_16**	MDPSPGVRFYVEFIWRSDGEVTIKFGLQFIIIVYPELTITFEIG ATWEGDSTFAIRVTFGFKVRVWPTIEFYFGFGVQWAEDNKIRFE FKAGIIIKLYDPLTLEFRFGVTWFGDSIVKVDFGVAITFRVYPT FEITFGVGATWQGDSKFAINIGVEFRFTVYPPAIITVGFGFTWE GDSTLKFGFYFGYTIE	0.044 unnamed protein [*Nesidiocoris tenuis*])
**TMB12_r1_17**	MSDSPGVAFYVKFEWRSDGTFRIEFGVTFKIIVYPDVIFQLTIG FIWEGDSKLKIQVTVGVEIRVWPTLAFYFGTGVRWTSENDVQID IQVGVKIKVYDPFIIDIRFGFTWRGDSKFEFTVGLTLQFRFYPT FTVNFGFGVTWKGDSKVTFEFGVEFRFRVYPPVEIDFGFGAIWF GDSFLQVGFYVGYKVK	0.05 unnamed protein [*Nesidiocoris tenuis*])
**TMB12_r1_18**	MSQSPGLKFYVTFRWNSDGKLQVEVGFKIEIFVYPTIFISLIFG FVWDGDSKLFIQISDGIEFQVWPDLSFYFGAGIRWSSSNKVEIE FKAGFKIRLYDPFEVIFTFGFTWKGDSTLAIQFGITFTFTFYPT FKIQFGFGVQWEGDSKVEFTFGFEIEVKVYPPVKFRFGAGVIWV GDSQLHVGFYVGYEIK	0.02 unnamed protein [*Nesidiocoris tenuis*])

Table S4. Experimentally tested designs

Design	Amino Acid Sequence	Expression	CD (DPC micelles)	SEC (DPC micelles)	Conductance	BLAST E-value*
**TMB10_163**	MRTSPGTKPYVKVRW<br>NTDNTVAVAFGAETD<br>YKLAPYLKTGVATET<br>EYNNSSLVKTGTEVK<br>TAYRLGPNAALETVV<br>RYNTDNTFGVEVAIE<br>YRLEPDLSVAPGTRW<br>NNSSLLAPYIKIKYK<br>LGPDLDVVTTIAYNT<br>DNTVGIETKVAYKTD	Yes	Negative maxima CD spectra at 215 nm	Mono-dispersed monomer	No	No match
**TMB10_164**	MRTSPGTKPYVKVRW<br>NTDNTVAVAVGAETD<br>YKLAPYLKTGVATEV<br>EYNNSSLTKAGTEVK<br>TAYRLGPNAALEVVV<br>RYNTDNTFGVEVAIE<br>YRLGPELSIAPGVRW<br>NNSSLLAPYLKIKYK<br>LGPDLDIVTTIAYNT<br>DNTVGIETKIAYKTD	Yes	Negative maxima CD spectra at 215 nm	Major monomer	No	0.011 (voltage-dependent channel)
**TMB10_165**	MRTSPGTKPYVKVRW<br>NTDNTVAVAFGAETD<br>YKLAPYLKTGVATET<br>EYNNSSLVKTGTEVK<br>TAYRLGPNAALEVVV<br>RYNTDNTFGVEVAIE<br>YRLEPDLSVAPGVRW<br>NNSSLLAPYVKIKYK<br>LGPNADIVTTIAYNT<br>DNTIGIETKIAYKTD	Yes	Negative maxima CD spectra at 215 nm	Major monomer	**Yes**	No match
**TMB10_166**	MRTSPGTKPYVKVRW<br>NTDNTVAVAFGAETD<br>YKLAPYLKTGVATET<br>EYNNSSLVKTGTEVK<br>TAYRLGPNAALETVV<br>RYNTDNTFGVEVAIE<br>YRLEPDLSVAPGVRW<br>NNSSLLAPYVKIKYK<br>LGPDLDVVTTIAYNT<br>DNTVGIETKIAYKTD	Yes	Negative maxima CD spectra at 215 nm	Mono-dispersed monomer	**Yes**	No match
**TMB12_0**	MQEKPGSAEGGTRTL<br>YNTDNTLKSGGYGVY<br>VLSPELVLFAAYFWN<br>NSSLQEFVAGAKYKL	Yes	Negative maxima CD spectra at 215 nm	Major monomer	No	No match

	SPYLETEVHLRYNTD NTFAVDVTTEGEYPV SPELKFRPGATYRWN NSSLNKLRPYLKVEY KLSPDLKGVVEVQYN TDNTVLVWFGATYKL SPDLEVTVMYGWNNS SLNWLLVDVKYKLSE					
**TMB12_2**	MQDRPGTLKVGGRTV YNTDNTFKSGGYAVY VLTPDLAGKTKYEWN NSSLQSFEFGGQYKL SPYARTEVSVQYNTD NTVKFRVVVEGEYPL SPNLRAFPGAEYVWN NSSLNKLFPYLRFEY DLSPELLGRLEFWYN TDNTFKVKLGAEYKL TPNLSVLVMYGWNNS SLQEFETETRYDLSS	Yes	Negative maxima CD spectra at 215 nm	Mono-dispersed monomer	No	No match
**TMB12_3**	MQDKPGSAKAGGWTT YNTDNTFKGGSYAKY VLSPNLALKGEYEWN NSSLNSFKAGAEYVA TPYLKTEVMTEYNTD NTFRVTVVTEGRYPV DPNLELFPGGWYTWN NSSLNKGAPYTRAEY KLTPDLKLLSQVVYN TDNTFKFDTGLEYKL SPNLKVKFEYGWNNS SLNEFTVQFEYDLSS	Yes	Negative maxima CD spectra at 215 nm	Mono-dispersed monomer	**Yes**	No match
**TMB12_4**	MQDKPGTVKGGGKGQ YNTDNTVKGGGYAVY TLSPDLSGKTEYLWN NSSLQELRVGARYRL SPYLETDVEVAYNTD NTFVLRVDTRGEYPL SPELKLFPGAKYAWN NSSLQKGSPYATFVY HADPNLLFKVTFRYN TDNTVEVEVGAEYKL SPNLSTFTAYGWNNS SLNWVRVETRYLFTY	No	N.D.	N.D.	N.D.	No match
**TMB12_5**	MQKKPGDLEGGTQVK YNTDNTFVFGGYAEY VLSPELSLRGKYDWN NSSLQVFRGGATYKL SPYLKVTFLGEYNTD NTARGAVVFEGEYPL	Yes	Negative maxima CD spectra at 215 nm	Mono-dispersed monomer	No	No match

	SPNLVLFPGAQYDWN NSSLNKGKPYLRVVY DLSPELKLVVEFWYN TDNTFKVFVGTKYKL SPNLELFVWYGWNNS SLNEFVADLRYQLTP					
**TMB12_6**	MEDKPGTAKGGGETK YNTDNTLDVGGYGEY VLTPELKGFTRYVWN NSSLQSLEVGATYKL SPYARVTVSVEYNTD NTAALKVTVEVFYPL SPNAFTVPGATYVWN NSSLQKGFPYTLFVY QVSPDLVLFVMVVYN TDNTLTLFFGGEYRL SPNLSTRTAYGWNNS SLNWVRTETRYDLSS	Yes	N.D.	Void (aggregate)	N.D.	No match
**TMB12_7**	METKPGSVWGGGEAK YNTDNTFEAGGYGEY HLTPNLTAFGQYMWN NSSLQKARGGLTYWL SPYARVTVFVEYNTD NTVTLFVVFEVFYPV TPELVLVPGAQYEWN NSSLNSLKPYLVVFY KLSPDLVGKVVFVYN TDNTFQVWFGAVYQL SPNLFVEVMYGWNNS SLNELKTRVEYLLSM	Yes	N.D.	Void (aggregate)	N.D.	No match
**TMB12_8**	MQDSKGSVRSGTEGE YNTDNTAKGGTYTTY VVTPDLEVTVKYEWN NSSLNRTEVGATYRV SPYARTTVSVKYNTD NTVKFDVTVEGDYPL SPNLKTHPGATYTWN NSSLNKLSPYTTVKY KLSPQLVGKVTFRYN TDNTVEVWFGAEYQL SPELVVEGAYGWNNS SLQKFKGRTEYQLTP	Yes	Negative maxima CD spectra at 215 nm	Mono-dispersed monomer	No	No match
**TMB12_9**	MQEKPGSLRAGTEFR YNTDNTFSLGFYAWY QLSPNLVGFAKYEWN NSSLNVFEAGAKYKL SPYLETVVSVVYNTD NTAKLKVRTESEYPL SPNLRLRPGADYEWN NSSLQKAFPYLTTEY	Yes	Negative maxima CD spectra at 215 nm	Mono-dispersed monomer, crystals diffracting at 8A.	**Yes**	No match

	KLSPDLVGKTTFVYNTDNTVKVVAGALYKLTPNLEVEFLYGWNNSSLNETVVRFDYVLTP					
**TMB12_tri_0**	MGSPGRTEVRGEGGYNTDNTFWGVVEFWYEASPNLSPYVRYKWGKSSKNVGWPGGRYKASPDAEVDVEVGYNTDNTVVFRVTLRFFYDASPNAKPYGEFTYEWGKSSKNKSMVYGGAKYKASPGAEVDFKGGYNTDNTFQGTVVFWYRLSPNAMGYGRYTWGKSSKNEFTGGGKYVGSE	Yes	N.D.	Major monomer	No	No match
**TMB12_tri_4**	MGSSQSPGRTETRTEGGYNTDNTVKAVTVTFYDLSPNAQPYVVYEWGKSSKNKGEPGGQYRLSPDAWVDVRVGYNTDNTVEFLVVFQFWYDASPDLKPYGEFWYKWGKSSKNKGAVYGGAKYRLSPNAEVEFKGGYNTDNTLEASVVFWYRLSPGAEGYGKYDWGKSSKNAFFGGGKYVGSE	Yes	N.D.	Mostly void (aggregate) but has monomeric peak	**Yes**	No match
**TMB12_tri_12**	MGSQGSPGRTEVTFEGGYNTDNTAWAATRFKYQASPNAEPYVEYKWGKSSKNVGWPGGKYKLSPDAETDTRTGYNTDNTLRVETETVFWYDASPDAKPYVRFRYVWGKSSKNKGAVYFGGKYKLSPNAEVEVEGGYNTDNTLEGRVEFRYRGSPNLEGYVVYLWGKSSKNEFTGGGTYVGSD	Yes	Negative maxima CD spectra at 215 nm	Mono-dispersed monomer	**Yes**	No match
**TMB12_tri_53**	MGSPGEGLVRTMVGYNTDNTVEVKTEFWYVLSPDAFPYLRYKWGKSSKNESSPGGTYRLSPDAETTAEAGYNTDNTLKVVVSTTFFYDASPNLKPYVTFTYKWGKSSKNKFEVYFGGEYK	Yes	N.D.	Mono-dispersed monomer	**Yes**	No match

	LSPNARSVTEVGYNTDNTAWAETKTEYQGSPNVKGVVEYFWGKSSKNQFWFGGEYRGSN					
**TMB12_tri_28**	MGSGDSPGVSEGRLKFGYNTDNTAWVEVEVWYWASPNLVPYVKYKWGKSSKNDFKPGGRYKLSPDAELDASGGYNTDNTLKADVQLDFWYDASPNLKPYVRFVYKWGKSSKNKFKVYFGGKYKLSPGAESEFEVGYNTDNTAEVRVRFKYRGSPNAWVVVQYDWGKSSKNDGESGVDYKGSE	Yes	N.D.	Monomeric and higher order oligomeric peaks	No	No match
**TMB12_tri_14**	MGSEDSPGRGETLVMTGYNTDNTAETRGETWYQLSPDFVPYLRYKWGKSSKNEFWPGGTYKLSPDAEVTFEVGYNTDNTLKFRVEFKVFYTATPDLHPYGEVKYEWGKSSKNKGAVYGGGKYRLSPNAEGDVQGGYNTDNTVELTFRFFYVGSPNLVGVVEYTWGKSSKNEFWFGGKYLGSD	Yes	N.D.	Mostly void (aggregate) but has monomeric peak	No	No match
**TMB12_tri_36**	MGSQPSPGQGESLVKVGYNTDNTADVTVEFWYVASPNLVPYAVYTWGKSSKNKFEPGAKYKLSPDAWVDVKVGYNTDNTAWAESVAEFWYKATPDLEPYVVFVYRWGKSSKNKFEVYFGGRYKLSPNAELDTVTGYNTDNTVVTTSQLDYWLSPNALLVVRYTWGKSSKNRVESGVKYKLSE	Yes	N.D.	Major oligomeric peak	No	No match
**TMB12_tri_32**	MGSGGSPGQTEVKTETGYNTDNTVDTVVEFWYVVSPDLVPYFVYFWGKSSKNKFEPGGQYKLSPDAWVDTRGGYNTDNTLEGKTVVEFWYDASPDAKPYVQFHYE	Yes	N.D.	Major monomer	No	No match

	WGKSSKNKFKVYFGG RYRLSPNAELDAATG YNTDNTVVTVVQFDY QGSPGLVGYVRYVWG KSSKNKFVFGGKYRG SE					
**TMB12_oval_1**	MGSESRQGSLGAYVR FVYNTDNTAEVGPGF EYEASPHLWVQVDVG WNNSSLVKFSVETSY KSPDGLEVKVGGEYN TDNTARGKVEVVFWY TLSPDLHPYGKVQYF WNNSSLNKGKPGGGF VYKLSPHAEFKFETG YNTDNTVEVHFWTDY KASPELEFSSGGVWN NSSLARTETRAKYKL TP	Weak	N.D.	N.D.	N.D.	0.003 (Signaling mucin HKR1 [*Mizuhopecten yessonsis*])
**TMB12_oval_2**	MGSQDKPGTAGGYFR AKYNTDNTAEAGPGG VYVLTPDLKLFVEFG WNNSSLFKLKVFADY KSPDGVDFRSGTEYN TDNTAETQFEVLFWY SVSPEFNPYGKFEYR WNNSSLNKAKPGGGA EYTFSPDLKVWTETG YNTDNTAETTVVVKY RLSPDLEVQTGTTWN NSSLVEFVTEVWYKA SD	Yes	N.D.	Major monomer	**Yes**	No match
**TMB12_oval_3**	MGSQPKPGDLGTYLE VEYNTDNTAKTGPGA VYVLSPHLAVETKSG WNNSSLLEVTASLKY ETPDGVVVEVGGTYN TDNTLDLFTDVEFWY TLSPHLHPYGRLRYE WNNSSLNKAQPGGGA LYVLSPHLRTRVDTG YNTDNTVKVKVETDY VVSPHVQVRVGGEWN NSSLFKTVVQFWYKL TE	Yes	N.D.	Major monomer, minor oligomer	**Yes**	No match
**TMB12_oval_4**	MGSQDSQGDLGGYLR VEYNTDNTAWVGPGG KYVLSPHAELDVAAG WNNSSLLWTEVAVVY WSPDGVQVKLGTRYN	Yes	Negative maxima CD spectra at 215 nm	Major monomer	**Yes**	No match

	TDNTVSTKLEVLFWY LLSPHLRPYGKTEYQ WNNSSLNKTRPGGGF QYDLSPHLATQFEAG YNTDNTAVAKVKTVY QLSPDAKVEGGTQWN NSSLVKFESQVDYKL SS					
**TMB12_oval_5**	MGSQDSQGDAGGYFR VEYNTDNTFWAGPGG KYALSPHLFLDVAAG WNNSSLLKFDFHVKY FSPDGLEVQVGFTYN TDNTLVFFVVVVFWY DLSPHVKPYGRLEYR WNNSSLNKFFPGGGA RYKLSPDLEVQADSG YNTDNTASTRFETVY KVSPEAEFKAGGQWN NSSLFKFQSQFDYKA SS	No	N.D.	N.D.	N.D.	No match
**TMB12_oval_6**	MGSQSNPGTLGAYFR AVYNTDNTFEAGPGF EYELTPDLHTQVDVG WNNSSLVKFTVETTY KTPDGVEFTFGGTYN TDNTVVLLVVVVFWY DLTPDLKPYGKTEYW WNNSSLNKVRPGGGF VYRLSPHLATRVDVG YNTDNTLFVKVSTKY DLTPHAEFEAGTEWN NSSLVRFEAVLKYKL SE	No	N.D.	N.D.	N.D.	No match
**TMB12_oval_7**	MGSEDKSGSAGSYLR VQYNTDNTVKLEPGS EYTLSPHLDTRVAVG WNNSSLASLKVSTVY RTPDGVEVEAGGEYN TDNTVDGVLRVQTQY QASPHLFPYTRTEYR WNNSSLNRSKPGGGF FYSVSPHLQFEVFVG YNTDNTFVVEVFTRY RVSPDVETKVGAAWN NSSLLSLVAETKYKL SE	Yes	N.D.	Major oligomer	No	No match
**TMB12_rect_1**	MGSQDKPGSAGGYAF VGYNNSSLIEFQAGA QYVITPHLKTDVRVG	Weak	N.D.	N.D.	N.D.	No match

	YNTDNTIKVEVKVKY KSPDGIEVEVKGEWN NSSLAATEVWAGAEY SLTPELHPYARAGYR WNNSSLNKPKGEAGA KYVLSPHARADVKTG YNTDNTIETEVTVQY QISPHAGGQVTVKWN NSSLVEVWVGGRYQL SE					
**TMB12_rect_2**	MGSQEKPGSAGGYAQ VGYNNSSLIKFEAGA QYVITPHLKVDLRVG YNTDNTIEVSLRTSY STPDGIDVSVEVQWN NSSLAAVEVQTGVWY SLTPHIHPYASAGYR WNNSSLNKPFAEAGL LYKLSPHAEAELRTG YNTDNTIRVELTVRY QLSPHLGTWAKTRWN NSSLVETWAGAKYQL SE	No	N.D.	N.D.	N.D.	No match
**TMB12_rect_3**	MGSQDKPGSLGAYAQ VGYNNSSLIEFQAGL EYVITPHLKTWLDAG YNTDNTAKGAVVVEY KTPDGIETRVRLEWN NSSLFKLDVKTGVMY SLTPHIHPYAAVGYT WNNSSLNKPKVEAGT KYVISPHLQVDVKVG YNTDNTIEFQVDVWY QVSPDAGGWASVKWN NSSLLEVWAGGKYQL SE	Weak	N.D.	N.D.	N.D.	No match
**TMB12_rect_4**	MGSSEQPGSAGAYAM TGYNNSSLIQTFVGG EYVITPHLKTRVETG YNTDNTARTEVETEY FSPDGAVAKVKAVWN NSSLAELLVVGGAQY FVSPDFFPYAMGGYK WNNSSLNKPVGVAGA KYRLSPHAWLKVEGG YNTDNTAELKVRAEY QISPEIGLLAQVDWN NSSLVEVKAGAKYKL SY	Yes	N.D.	Major monomer, minor oligomer	**Yes**	0.038 (multicopper oxidase domain-containing protein [*Verrucomicrobiaceae bacterium*])
**TMB12_rec**	MGSSEVPGSAGGYSK	Yes	N.D.	Mostly	No	No match

**t_5**	TGYNNSSLARTETGA DYVLSPHLVVQVAVG YNTDNTAELVVRVKY ETPDGIEFEVEVVWN NSSLIKFRAMTGAWY SLSPHLFPYAKAGYV WNNSSLNRPQAFAGA QYLLSPDLWLDVFGG YNTDNTIKLFVRVQY WLSPDLGTEAEVVWN NSSLVWVFAGAKYKI SD			void (aggregate), has monomeric peak		
**TMB12_rect_6**	MGSQPKPGSAGGYAA VGYNNSSLAKVEVGA EYQISPHAHVRLSTG YNTDNTAETVLVVVY ESPDGLRVKVVLRWN NSSLVQVEVMAGGLY VITPHLKPYSMAGYV WNNSSLNKPKGDGGA DYDISPDAQTKVSVG YNTDNTVELVVRVEY RISPDAGGFVEVKWN NSSLVEVKVGGKYKI SW	No	N.D.	N.D.	N.D.	No match
**TMB12_rect_7**	MGSSSRPGSIGLYAE TGYNNSSLAKTDAGG VYQISPHLKTWFAVG YNTDNTFSGRVQTKY DDPDGFHVSVKTEWN NSSLVSFEVKAGAMY QISPHAQPYAATGYQ WNNSSLNSPFVEVGA RYWISPHAFVDVAVG YNTDNTFVLFVTVFY QVSPDAGGWATGKWN NSSLAETWAGGEYVI DP	Weak	N.D.	N.D.	N.D.	No match
**TMB12_rect_8**	MGSEKKPGSAGAYAR AGYNNSSLIVGDAGG KYVISPHLETEVRVG YNTDNTAKLVVKVVY ETPDGAFVEVEVVWN NSSLISVVVSAGAWY DLDPHIKPYAQAGYQ WNNSSLNKPFAWAGA RYFISPDLEVDTATG YNTDNTFKLKTAVKY RLSPEAGLRFETVWN NSSLVEVWAGTEYKL SE	Yes	Negative maxima CD spectra at 215 nm	Mostly void (aggregate), has monomeric peak	**Yes**	No match

**TMB12_rect_9**	MGSQKKPGTAGTYAETGYNNSSLAVTRGGTRYVISPHAELDVSGGYNTDNTAFATVTVDYWTPDGIEFKVTVTWNNSSLAKVVVEAGAWYDLSPDAKPYVKGGYEWNNSSLNKPFGKAGAEYKVSPDLKVQVELGYNTDNTFFLRVQVDYKLSPHLGLKVETNWNNSSLAETWVGAEYQLTP	Yes	N.D.	Mostly void (aggregate), has monomeric peak	No	No match
**TMB14_1**	MGSESEKDEDVGVEYKKYDNQETEKRVYAKQKKSDKNELGVDYKKKSDNKTEKSVRYKYRYDDKLEVKVEYKKDSDDKDDLKVEVEYKRSDNAKLKAQYDKDKSDKWKVDVGGDYRIKKSEKSSLTPGGKLDVDKSRKVKLEPYARYEYKMSERSKADVELKADSDDKKQVDLKYSYKDSDKSKVDVKASFQKDSKWDAGVEVRYKKS	No	N.D.	N.D.	N.D.	0.0005 (oligogalacturonate-specific porin KdgM family protein [*Kluyvera ascorbeta*])
**TMB14_2**	MGSKSEKSSSVGVEYSKKDDQDTEKRVYAKQKKDKDNELGVEYKKKSDDKTEKKVDYKYKYSKELEVKVDYKKDSDDKDELYVEVDYKRSSKAYLKAKYRKDDKDDWSVSVGGKYEIKKSEKSYLEPGGELTVDDKNKVDLRPDARYKYKESKDSEADVRLEAQDKDRKSVELEYRYRTDDKSDVRVKADFDKDSQYKAGVEVKYDDD	Yes	N.D.	Mostly void (aggregate), has monomeric peak	**Yes**	No match
**TMB14_4**	MGSKQEKDTKVGVRYQKKKDDDTELDLEAKQRKDDKLELGVRYKYKSDSSWSLEVDLKYTESDKLDVELSYEYKDDSKKSLDLKVDYKRSKDAKLSASAKAEDDSKWSVSVGGQYRIKQSEKSYLEPGGSVDVDS	No	N.D.	N.D.	N.D.	No match

	SNKVDLKPYVRYRYK RSEKSEAGVEFGKSD KDKKEVKFDYKYEQD SRSYLDLEYRKDKDD DSEWGFKYEYDDS					
**TMB14_5**	MGSDSSSKEDVSVKY SKDKRNKSRVSVEVK QEQDSSNSLKVRYDK EDDDKSSVELGYEQD DDDKNKLKADVRKDK DDDVELEARYEYKRD QYLYLEAQYKKKKDS KYSLNVGGYYKIPLA DKLEVEPGGDVKYDD DSKKKLSPYVRVKYQ KDSNAEAGAEYKADE DKKSKLKLKLRYKED KDADVELEYEKDSDD KSEVGVRVKYRKD	Yes	N.D.	Void (aggregate)	No	No match
**TMB14_6**	MGSSDKKDDELEVEY SKDDKSKKSLSVKLK YKQDKDLDLEVEYRK DSDDKNDLRLGLKYR EDKKLELEASVKKDK DNKVELKAELRYKRD SYLKLYASAKARDDS KSELEVGGDYKVPYS DKLYLEPGGSVKYYD DSKSELKPYVKAEYK TDKDAKAEADYEAKE DDKKELKVRLDYKED KDSEVSLKYRADKDN RKEVGVKYKYKKD	No	N.D.	N.D.	N.D.	No match
**TMB14_7**	MGSESDSKERLRVEY KQDSDNENEVSVELE QKQDSSLDLRVEYKQ KDDDRNSVKLGLDYK QDSRLKLYAEVEYYK DQSVELEAQLEYKED KDLELYAKYKYKDDS KWTLEVGGKYKEPKA KDLEVEPGGSVDYYK DSKSKLNPSVRLEYK EDDKAYADAYLQADD KSQWKLDVKLKYKLD KDAEVDLKYSADDKN KNSVGVELTYKKE	No	N.D.	N.D.	N.D.	No match
**TMB14_8**	MGSREKKDTKVSVKY SKDDKDDSETSVRLE YKEDDKLALGVQYKK	Yes	Negative maxima CD spectra	Major monomer	**Yes**	0.012 (unnamed protein

	KSDDSSSTKVDLRYD<br>SDDKLKLEAAVEKKD<br>DSRTETEAKLEYKED<br>SYLKLYAKYKKDSDD<br>KYSLEVGGKYRVPKA<br>KDLKVEPGGSVEYYK<br>DSKYKVKPSVRLEYK<br>TDKDARAGVYLEADS<br>DKSWKLTADLEYKLD<br>YNSRLRLEYEADDKN<br>KSSVGVELEYKYD		at 215 nm			product<br>[*Psylliodes chrysocephala*])
**TMB14_9**	MGSKEKKKTKVEVSY<br>YKDDDDQSRTSVRLE<br>YDLDKDLKLGVEYTK<br>KDDDSSSTQVRLEYK<br>QDKKLDLEASVQKKK<br>DSDTRTSAELKYKRD<br>SYLDLYAKYEKKSDD<br>RYRLEVGGKYSVPLA<br>KDLKVDPGGSVEYYK<br>DSKKKVSPSVKLRYR<br>KDDKADAGVDLRAYE<br>DSSWKLKAYLKYKED<br>KKSSLELEYEADSKN<br>KSQVGVKLRYEED	No	N.D.	N.D.	N.D.	No match

*Protein BLAST search was performed against the non-redundant (nr) protein sequences database. The lowest E-value corresponding to a naturally-occurring protein is given. Many designs were matched with high confidence to previously-published *de novo* designed 8-stranded TMBs with < 50 % sequence identity.

Supplementary Figures

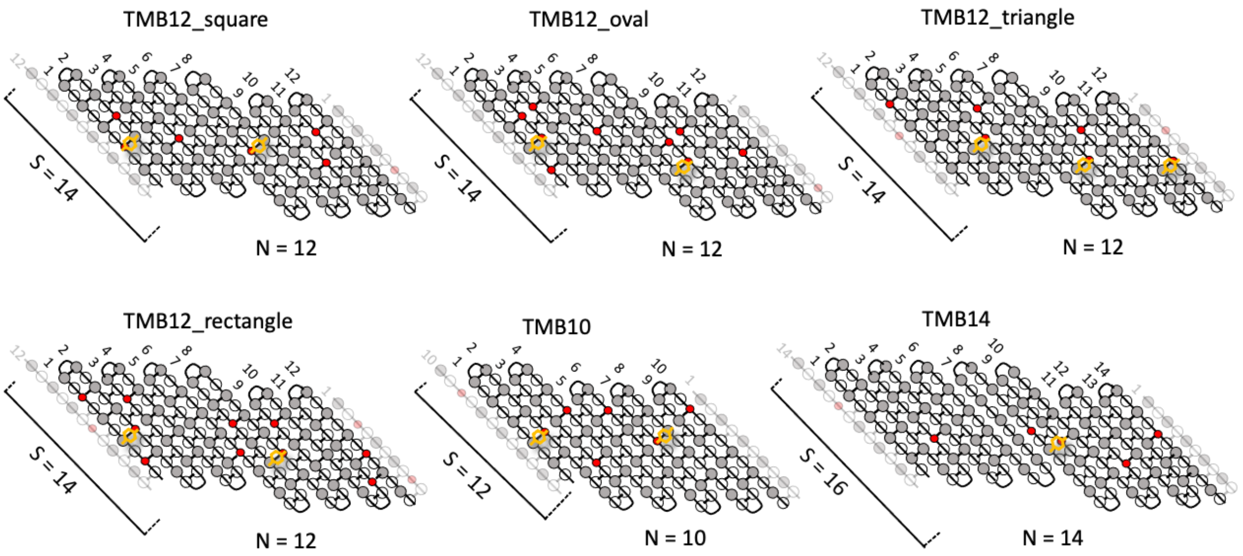

Figure S1: Six new β-barrel blueprints were generated here: one β-barrel of 10 strands (N=10) and a shear number of 12 (S=12), one β-barrel of 14 strands (N=14) and a shear number of 16 (S=16) and three β-barrels of 12 strands (N=12) and a shear number of 14 (S=14). The residues facing the β-barrel lumen and surface are shown as gray and white circles, respectively. Glycine kinks are shown as red circles and are facing the lumen. The tyrosine residues belonging to the Tyr-Gly-Asp/Glu folding motif are shown in orange.

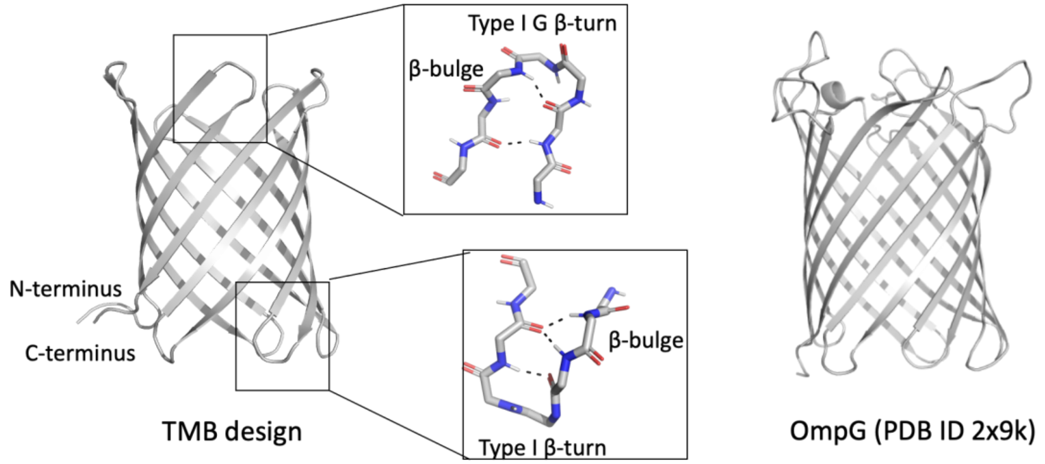

Figure S2: The β-strands of *de novo* designed TMBs are connected with short β-turns on both sides of the barrel: *cis*-hairpins (N- and C-termini side) are connected with canonical type I β-turns preceded by a β-bulge; *trans*-hairpins are connected with type I β-turns directly followed by a G-bulge. By comparison, naturally-occurring TMBs (exemplified here by OmpG, right) feature mostly long, disordered, loops on the *trans* side.

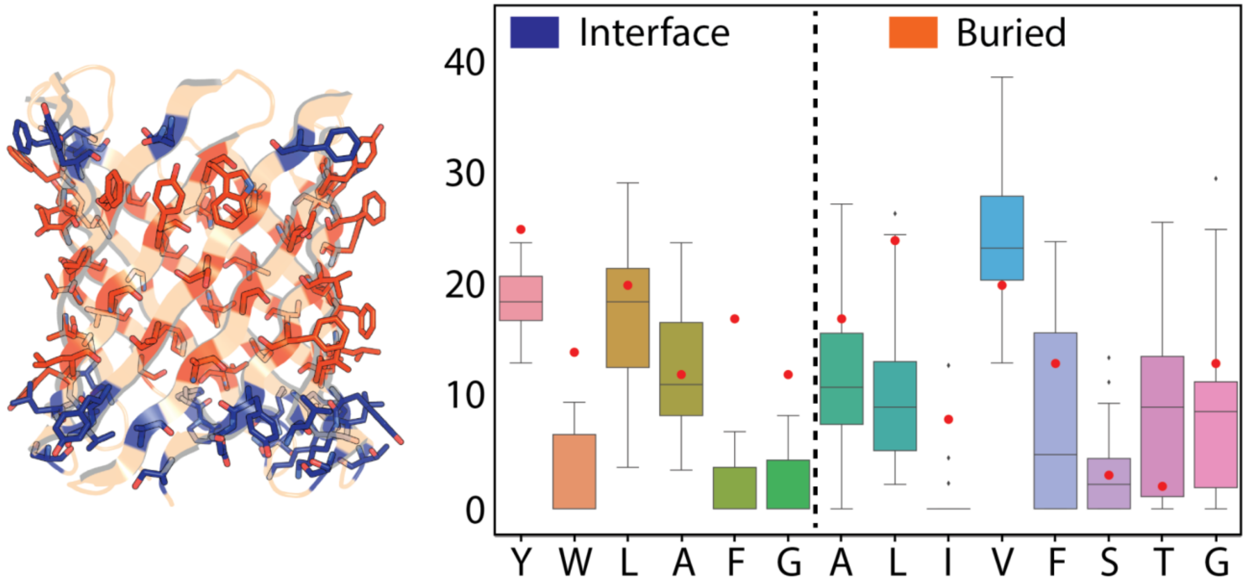

Figure S3: Amino acid composition of the membrane exposed surface of designed beta-barrel pores. Y-axis is calculated composition among all types of amino acids in the interface and buried region for each design respectively. The red dots are averaged amino acid compositions for the indicated amino acids in the respective regions over all transmembrane beta-barrel proteins in the OPM (Outer Membrane Protein) database.

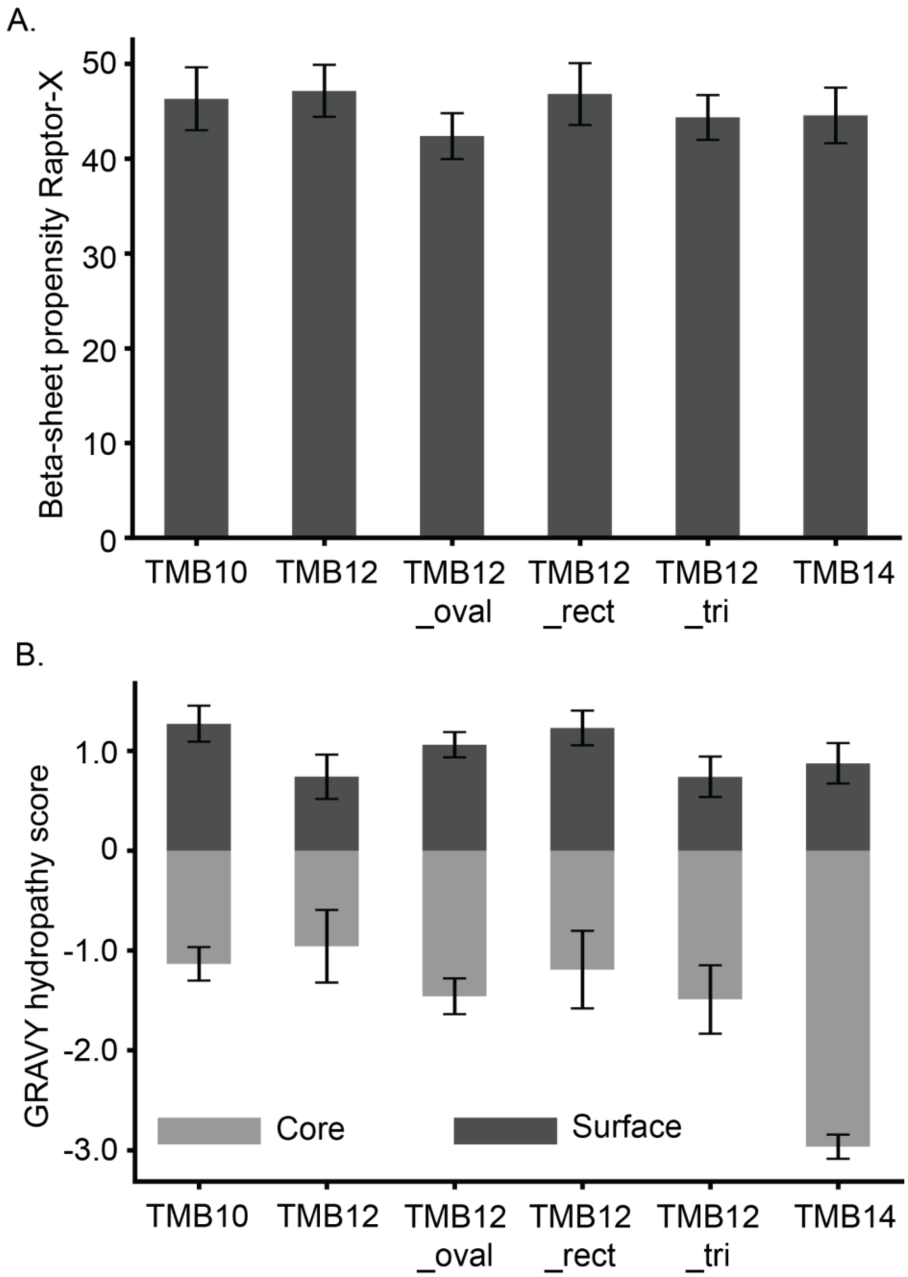

Figure S4: A. Predictions of beta-sheet propensities using RaptorX for different designs. B. GRAVY hydropathy values for the different types of designs and their differences between the pore lining core and surface exposed residues.

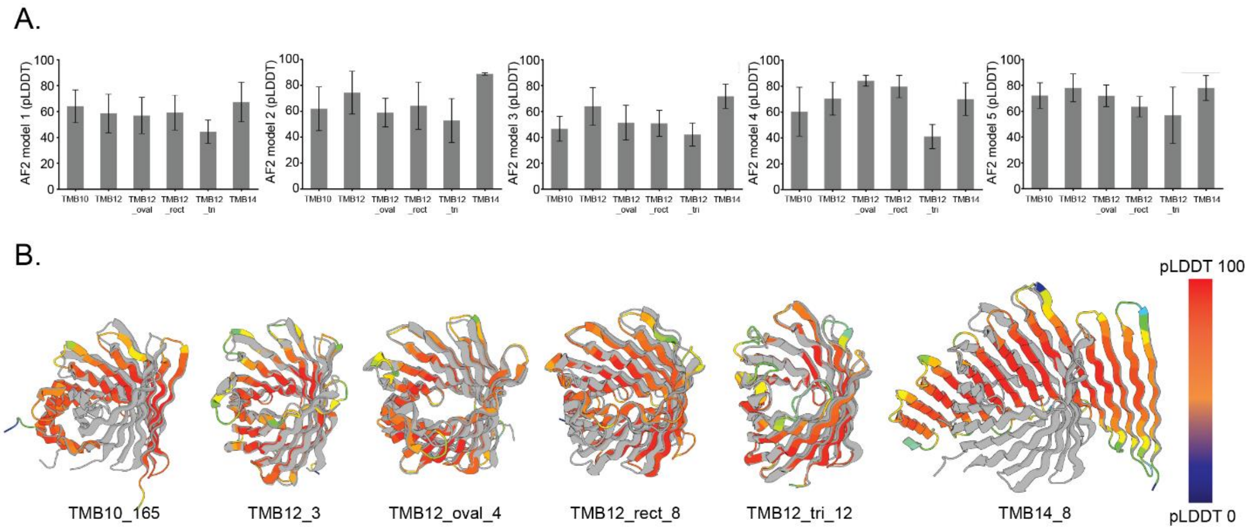

Figure S5: A. Mean pLDDT values for the different types of designs predicted from single sequence without MSA using Alphafold2 and the 5 models. B. Best predicted structures from Alphafold2 (shown as spectrum) are aligned to the design models (shown in gray) for each type of pore. The Alphafold2 structures are colored by per residue pLDDT values.

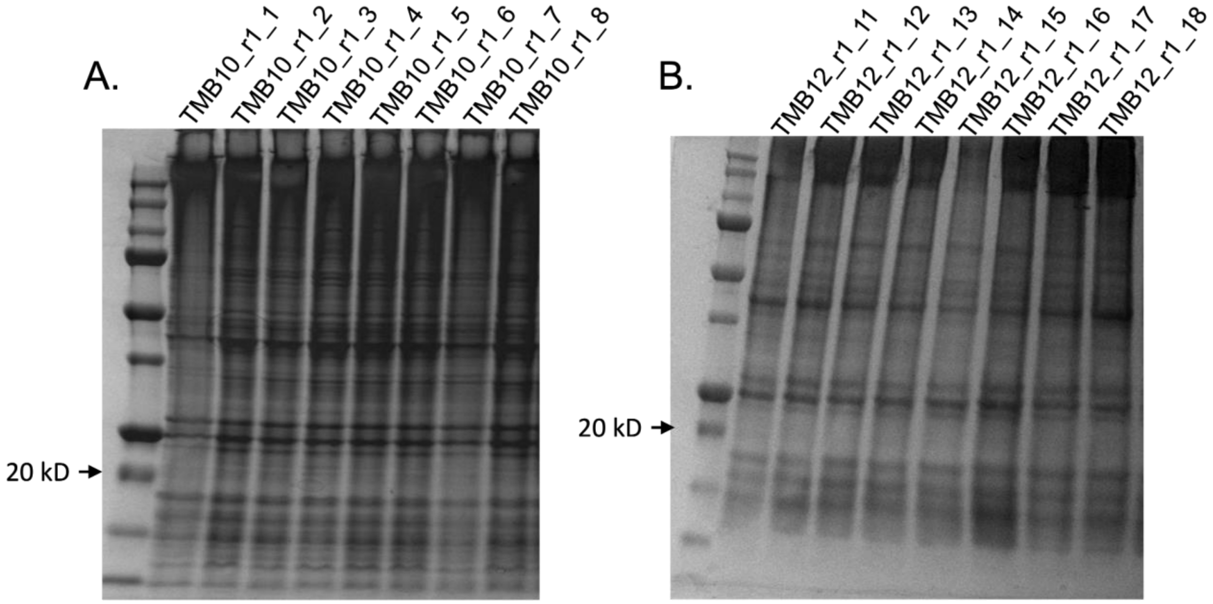

Figure S6: Coomassie stained SDS-PAGE gels showing the first 16 “optimal” 10- and 12-stranded TMB designs which failed to express. The expected molecular weights of the designed proteins were around 22 kDa for TMB12 and 18 kDa for TMB10 designs.

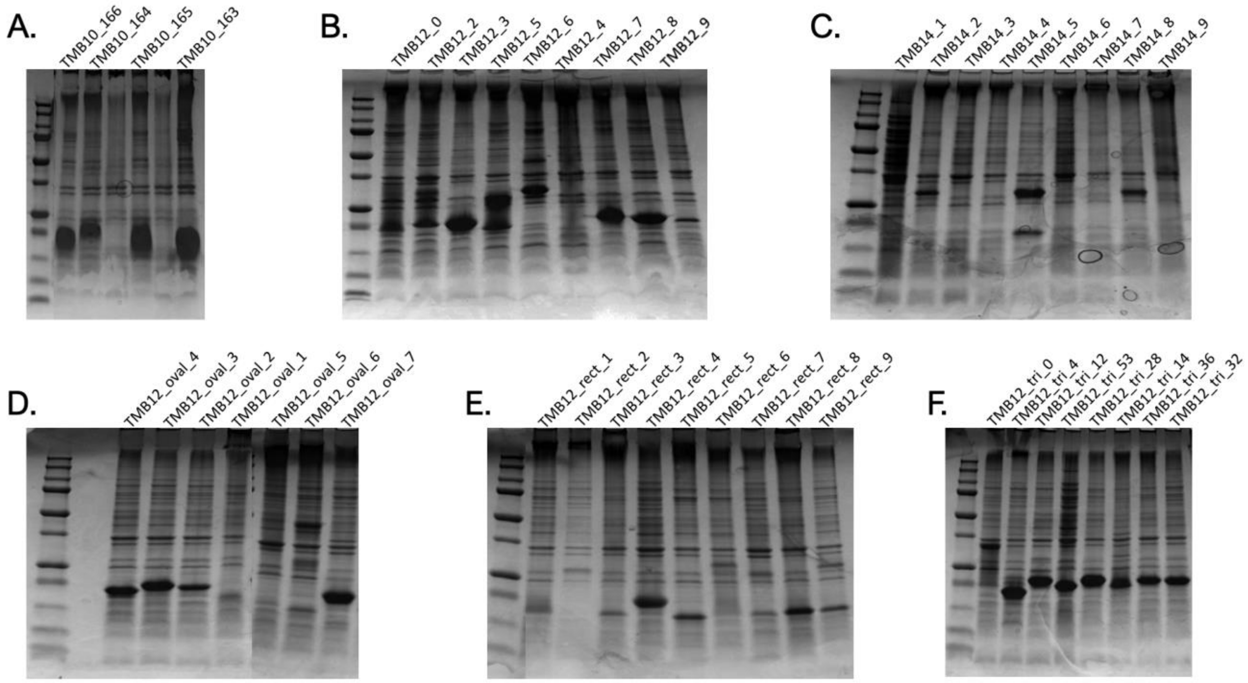

Figure S7: Coomassie stained SDS-PAGE gels showing expression bands for the different designs from insoluble fractions of corresponding lysed cell pellets.

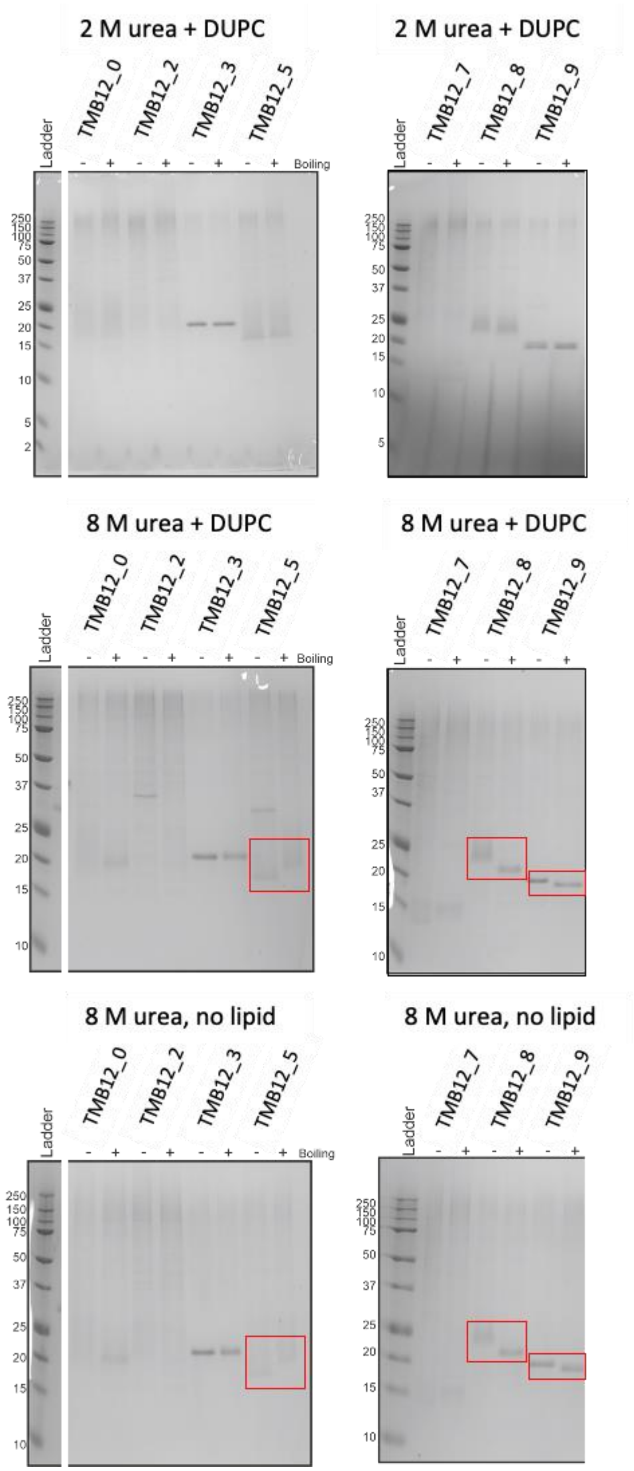

Figure S8: De novo designed TMBs do not exhibit the heat-modifiable behavior on cold SDS-PAGE gel characteristic of folded natural TMBs. No shift in band positions were observed after boiling the samples refolded in DUPC large unilamellar vesicles (LUVs). Band shifts after boiling (red rectangles) were observed only in conditions where the native β-barrel fold can not form (8 M urea with or without lipids).

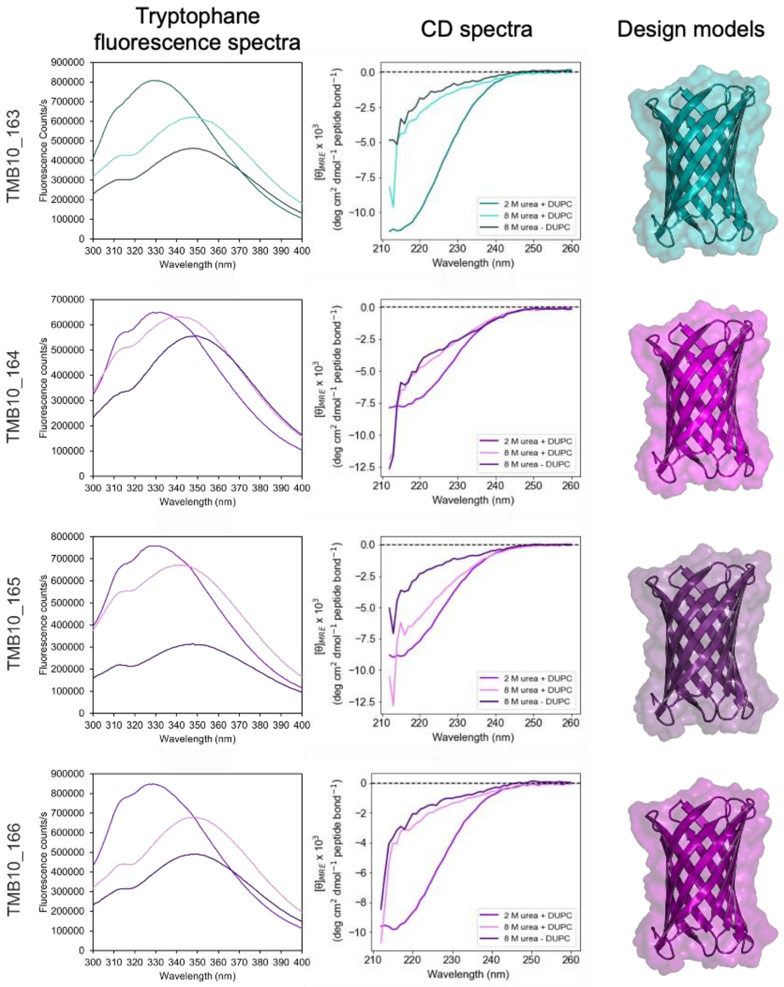

Figure S9: Biophysical characterisation of TMB10 designs (right: design models) for folding in DUPC LUVs. Tryptophan fluorescence spectra (left) and far-UV CD spectra (center) are shown for re-folded proteins in the presence of LUVs and 2M urea (allows TMB folding while reducing aggregation in water), in 8 M urea in the presence of LUVs and in 8 M urea in the absence of lipids. TMB10_163 was selected for further characterization (teal), as it demonstrated a clear fluorescence λ_{max} shift and change in β-sheet structure content between 2 M urea, 8 M urea, and no lipid conditions.

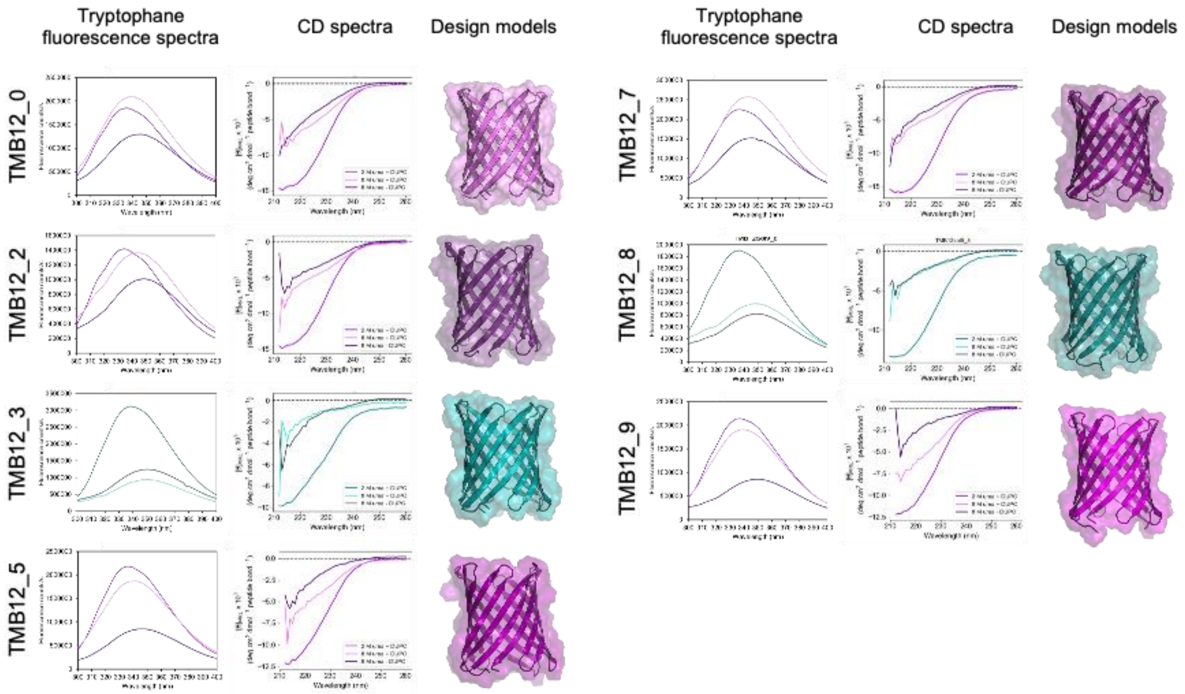

Figure S10: Biophysical characterisation of TMB12 designs with a square shape (right: design models) for folding in DUPC LUVs. Tryptophan fluorescence spectra (left) and far-UV CD spectra (center) are shown for re-folded proteins in the presence of LUVs and 2M urea (allows TMB folding while reducing aggregation in water), in 8 M urea in the presence of LUVs and in 8 M urea in the absence of lipids. TMB12_3 was selected for further characterization (teal), as it demonstrated a clear fluorescence λ_{max} shift and change in β-sheet structure content between 2 M urea, 8 M urea, and no lipid conditions. Similar spectra are observed for the TMB12_9 design, suggesting that the design is also folding into a TMB structure.

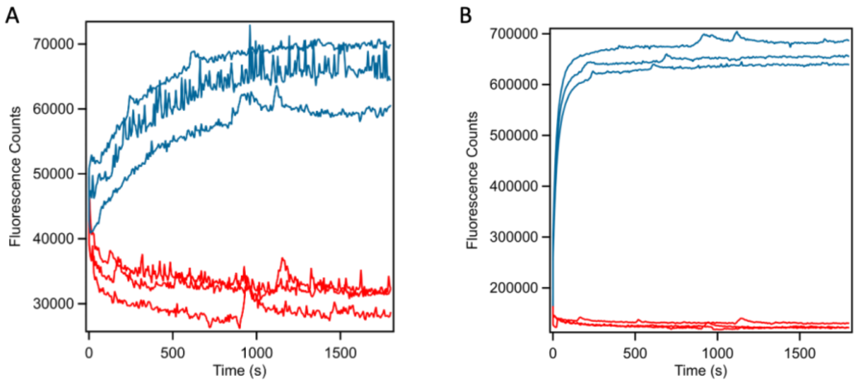

Figure S11: Folding kinetics of designs TMB10_163 (A) and TMB12_3 (B) in DUPC (blue lines) and DMPC (red lines) LUVs at 30°C and monitored by intrinsic tryptophan fluorescence. The difference in folding rates associated with the length of the lipid chain is consistent with intramembrane folding.

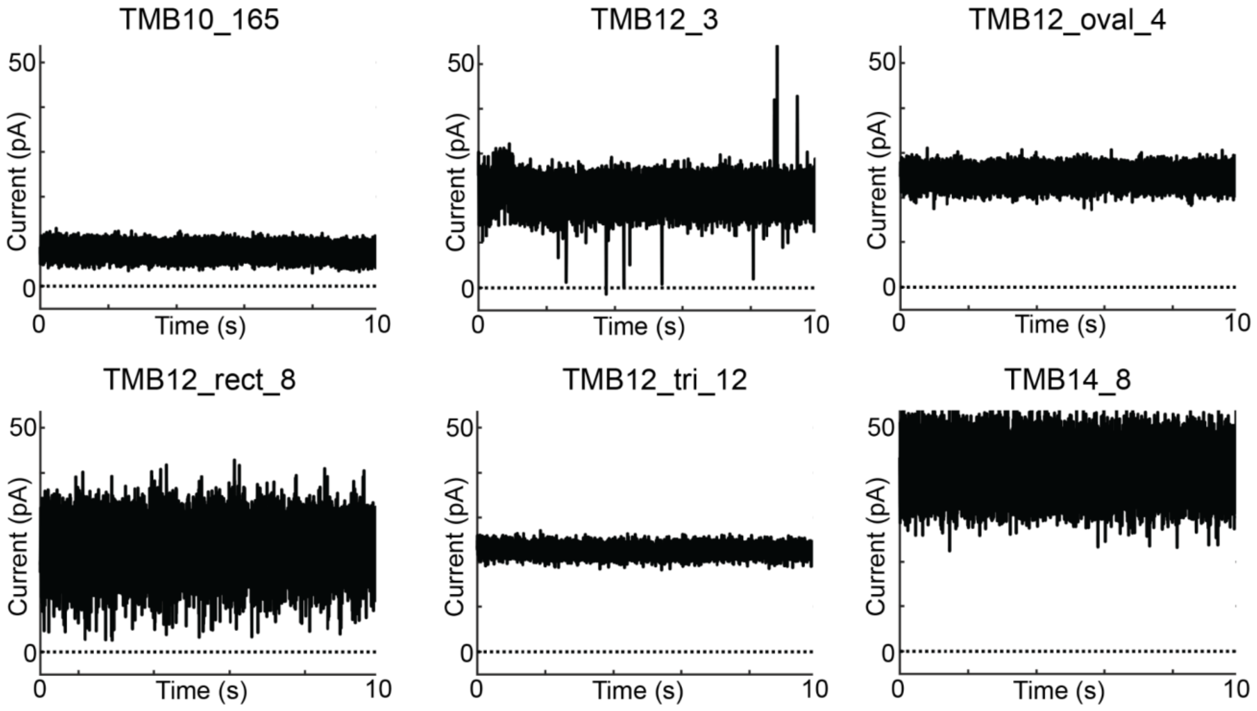

Figure S12: Raw unfiltered current traces of one example for each different type of pores recorded at 5kHz sampling rate. Applied voltage is 100 mV and the cis and trans buffer for all conditions is 500mM NaCl. Different noise levels are a result of the different bilayer capacitances at the time of recording and noise from adjacent cavities in the MECA recording chip from Nanion. 10s reads show characteristics of stable non-gating pores in the membrane.

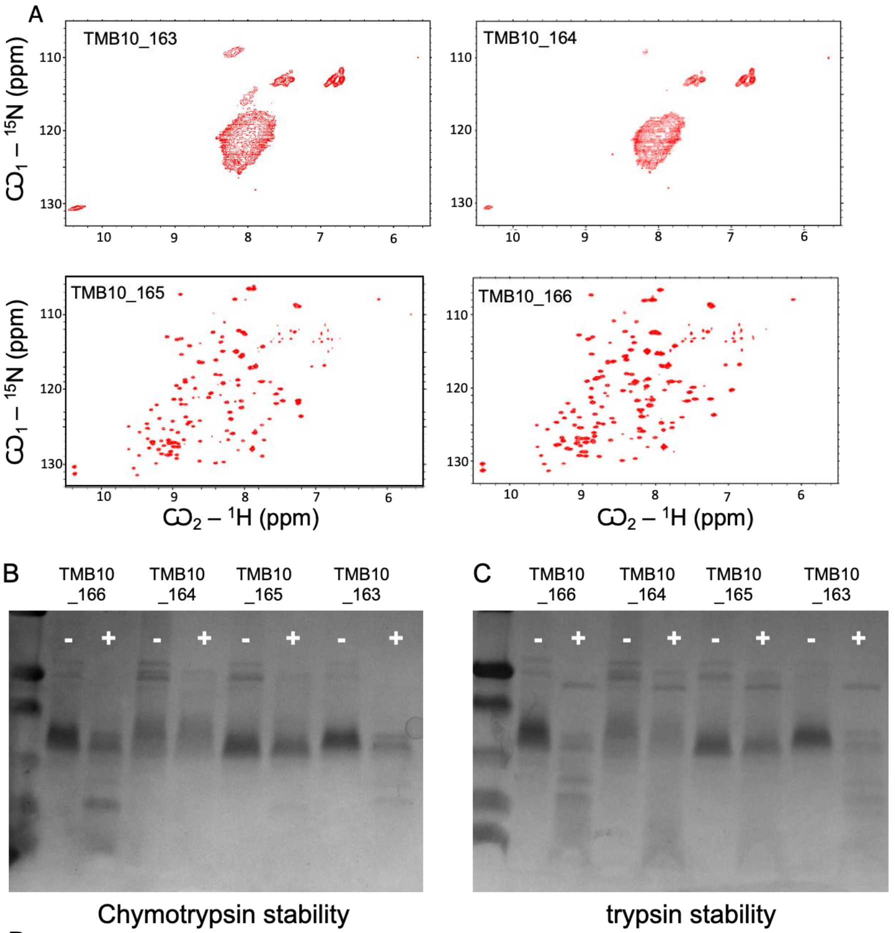

D

```
TMB10_163 MRTSPGTKPYVKVRWNTDNTVAVAFGAETDYKLAPYLKTGVATETEYNNSSLVKTGTEVK 60
TMB10_164 MRTSPGTKPYVKVRWNTDNTVAVAVGAETDYKLAPYLKTGVATEVEYNNSSLTKAGTEVK 60
TMB10_165 MRTSPGTKPYVKVRWNTDNTVAVAFGAETDYKLAPYLKTGVATETEYNNSSLVKTGTEVK 60
TMB10_166 MRTSPGTKPYVKVRWNTDNTVAVAFGAETDYKLAPYLKTGVATETEYNNSSLVKTGTEVK 60

TMB10_163 TAYRLGPNAALETVVRYNTDNTFGVEVAIEYRLEPDLSVAPGTRWNNSSLLAPYIKIKYK 120
TMB10_164 TAYRLGPNAALEVVVRYNTDNTFGVEVAIEYRLGPELSIAPGVRWNNSSLLAPYLKIKYK 120
TMB10_165 TAYRLGPNAALEVVVRYNTDNTFGVEVAIEYRLEPDLSVAPGVRWNNSSLLAPYVKIKYK 120
TMB10_166 TAYRLGPNAALETVVRYNTDNTFGVEVAIEYRLEPDLSVAPGVRWNNSSLLAPYVKIKYK 120

TMB10_163 LGPDLDVVTTIAYNTDNTVGIETKVAYKTD 150
TMB10_164 LGPDLDIVTTIAYNTDNTVGIETKIAYKTD 150
TMB10_165 LGPNADIVTTIAYNTDNTIGIETKIAYKTD 150
TMB10_166 LGPDLDVVTTIAYNTDNTVGIETKIAYKTD 150
```

Figure S13: Relative stability of TMB10 designs (163-166). (A) Designs TMB10_165 and TMB10_166 feature well dispersed NMR ^{1}H-^{15}N HSQC spectra. (B-C) Trypsin and chymotrypsin challenge reveals differences in stability between designs, with TMB10_165 showing the highest stability to both proteases. (D) The designs differ only by 2-9 residues on the lipid-exposed surface, introduced using Rosetta.

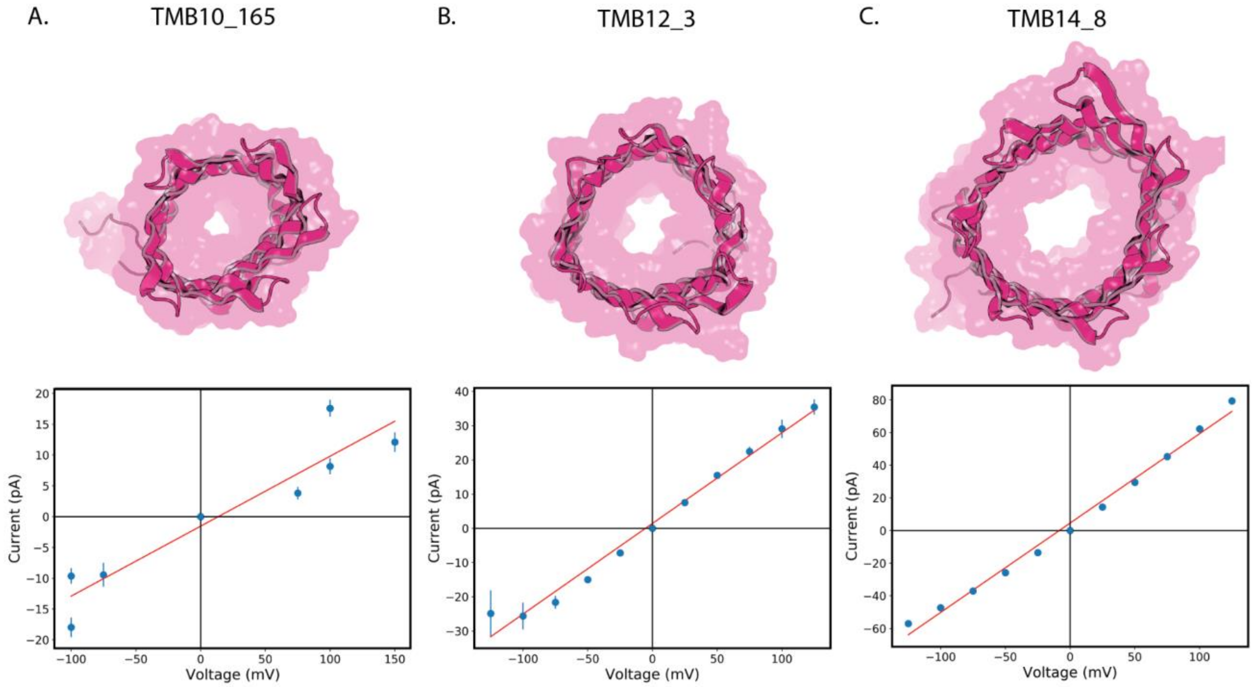

Figure S14: Current vs Voltage plots for three designs pertaining to TMB10_165, TMB12_3 and TMB14_8 pores. All measurements were carried out in 500mM NaCl solution (symmetric across bilayer).

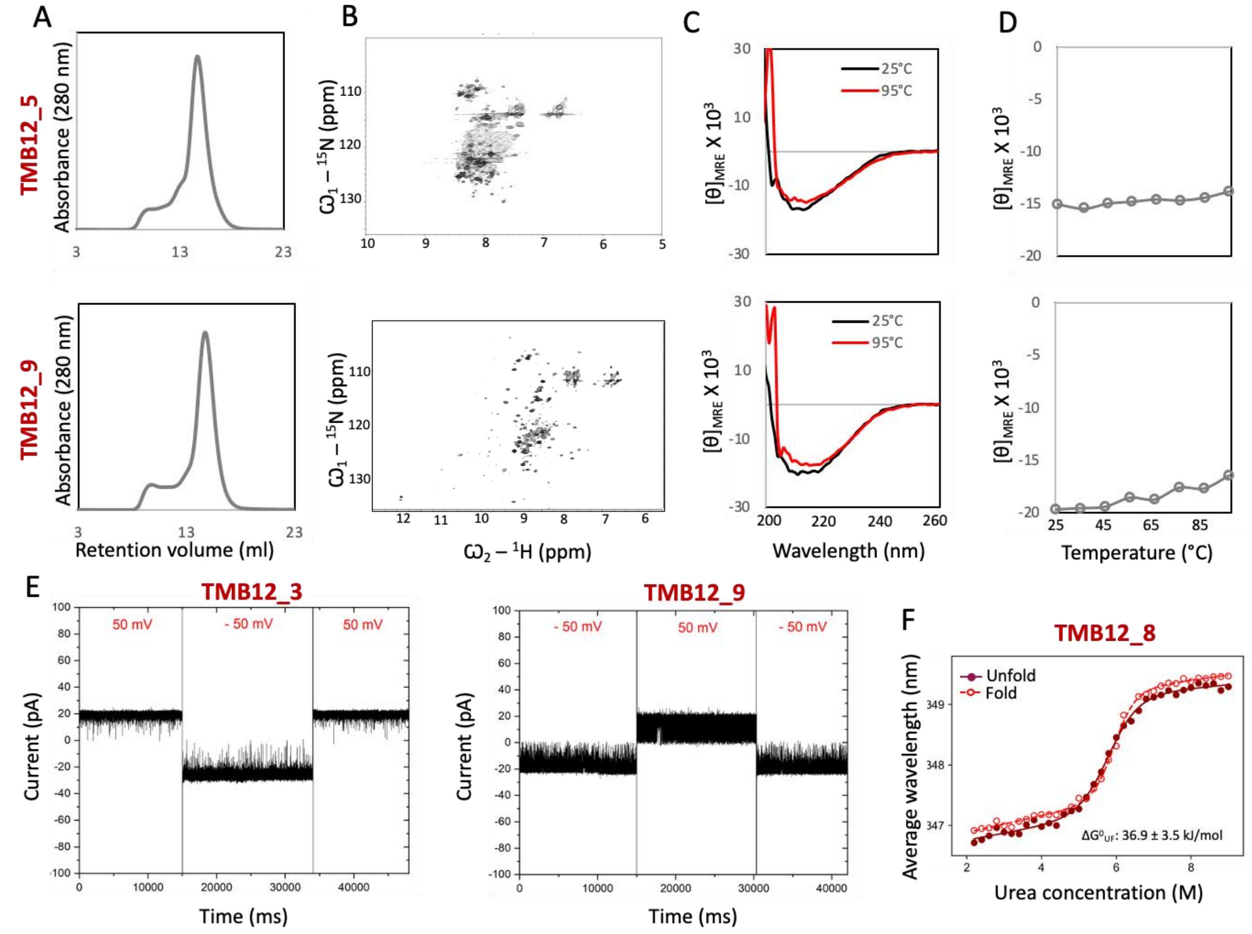

Figure S15: Designs TMB12_5 and TMB12_9 both feature monodispersed SEC elution profiles consistent with a monomeric TMB12 (A) and far-UV CD spectra characteristic of β-sheet proteins in DPC micelles (C) that remain stable up to 95°C (D). However, only TMB12_9 has a dispersed NMR ^{1}H-^{15}N HSQC spectrum indicative of a folded TMB (B). Stable nanopore activity was observed for designs TMB12_3 (stable signal) and TMB12_9 (gated signal suggesting lower stability), but not for TMB12_5 (E). TMB12_8 cooperatively and reversibly folds/unfolds in DUPC LUVs with a similar Cm^F to TMB12_3 (5.7 ± 0.3 M) but with a less sharp transition and hence lower unfolding free energy (F).

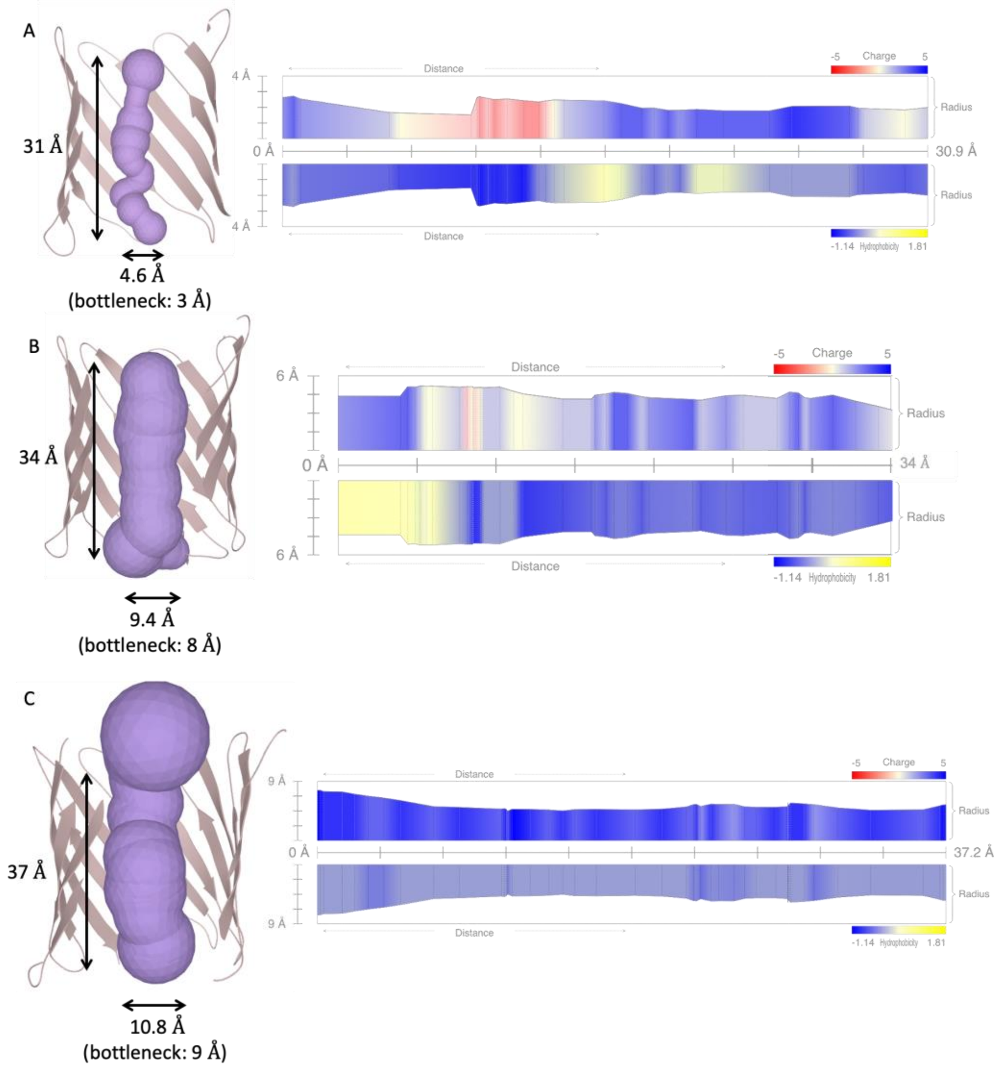

Figure S16: MOLE 2.5 pore size calculations (left), charge and hydrophobicity profiles (right) for designs TMB10_165 (A), TMB12_3 (B) and TMB14 (C).

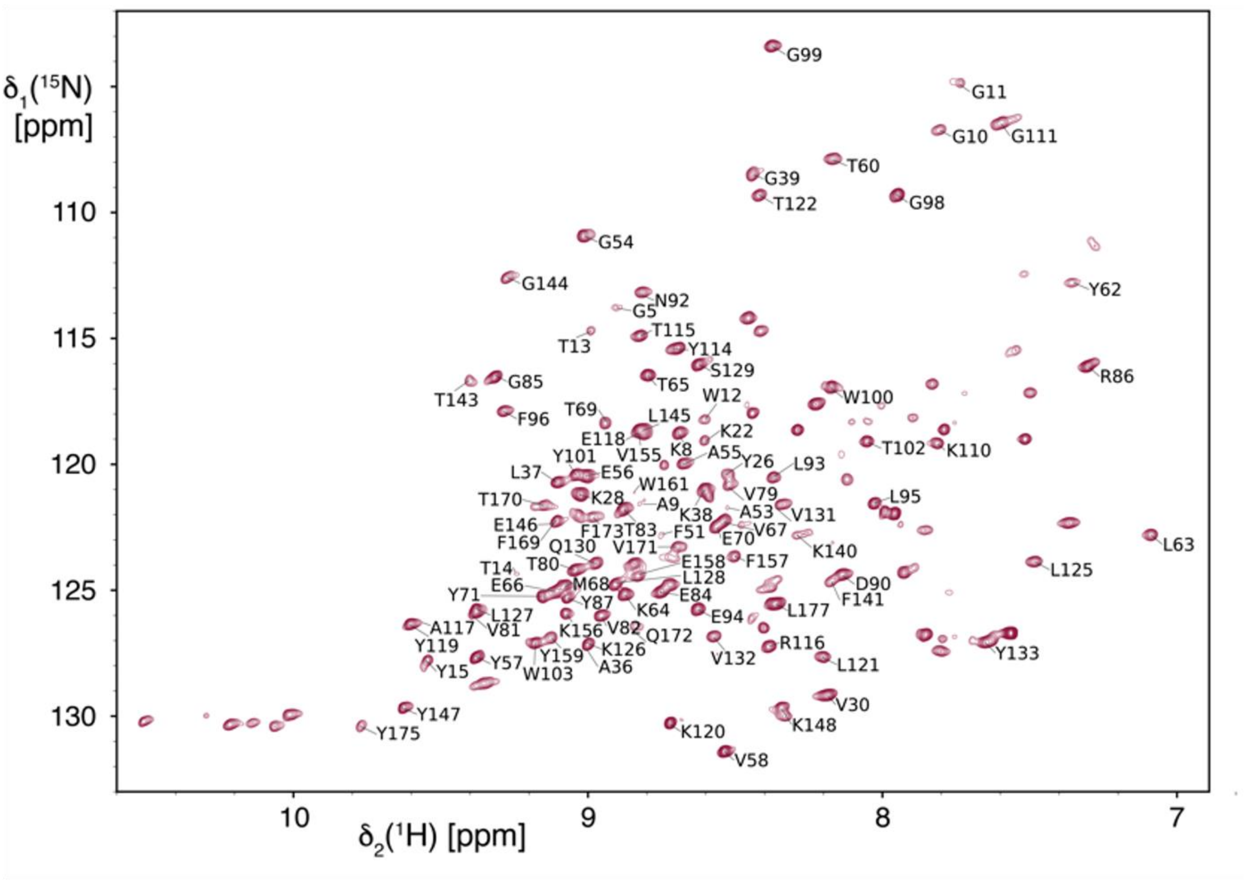

Figure S17: 2D [15N,1H]-TROSY NMR spectrum of [U-2H,15N]-TMB12_3 in LDAO micelles with sequence-specific resonance assignments.

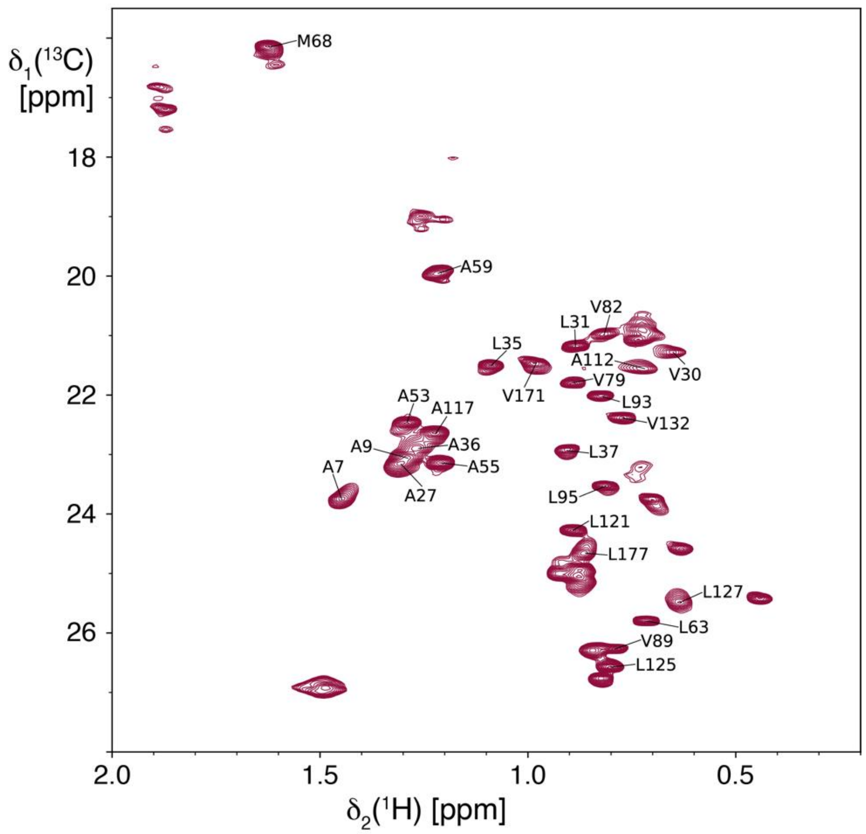

Figure S18: 2D [^{13}C,^{1}H]-HMQC of AMLV-^{1}H^{13}C-methyl-labelled TMB12_3 in LDAO. Sequence-specific resonance assignments are indicated.

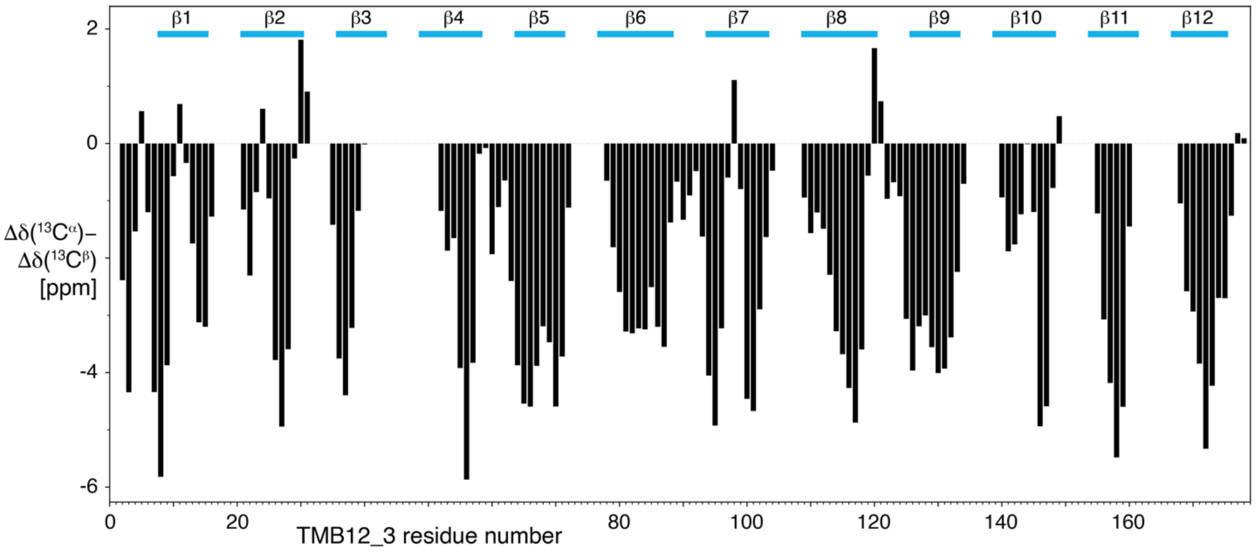

Figure S19: Secondary chemical shifts of TMB12_3 from sequence-specific resonance assignments. Consecutive stretches of large negative values indicate the presence of β-strand secondary structure. The positions of the 12 β-strands are indicated by blue lines.

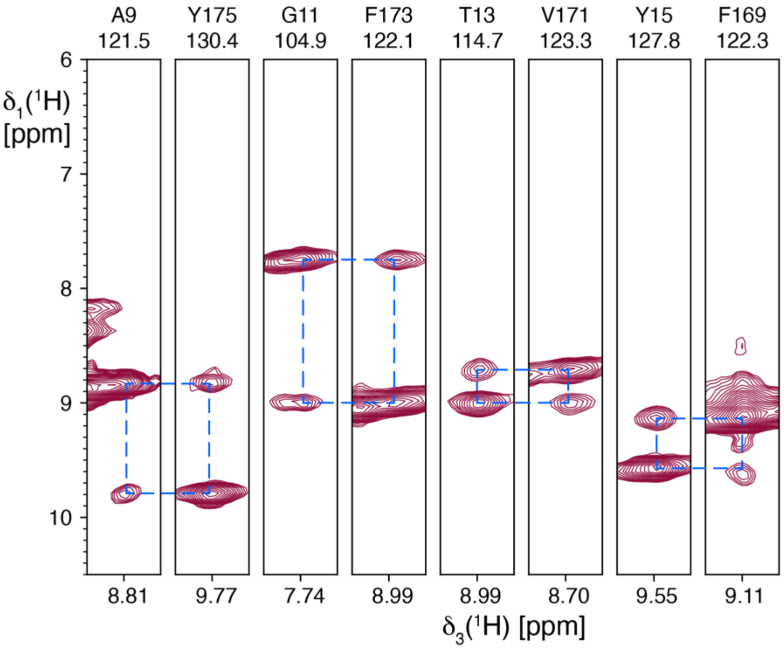

Figure S20: Strips from the 3D [^{1}H,^{1}H]-NOESY-^{15}N-TROSY experiment of TM12_3 in LDAO micelles. Strips were taken for the residue pairs involved in the antiparallel β1–β12 pairing. The NOE cross peaks are connected to the diagonal peaks by blue dashed lines.

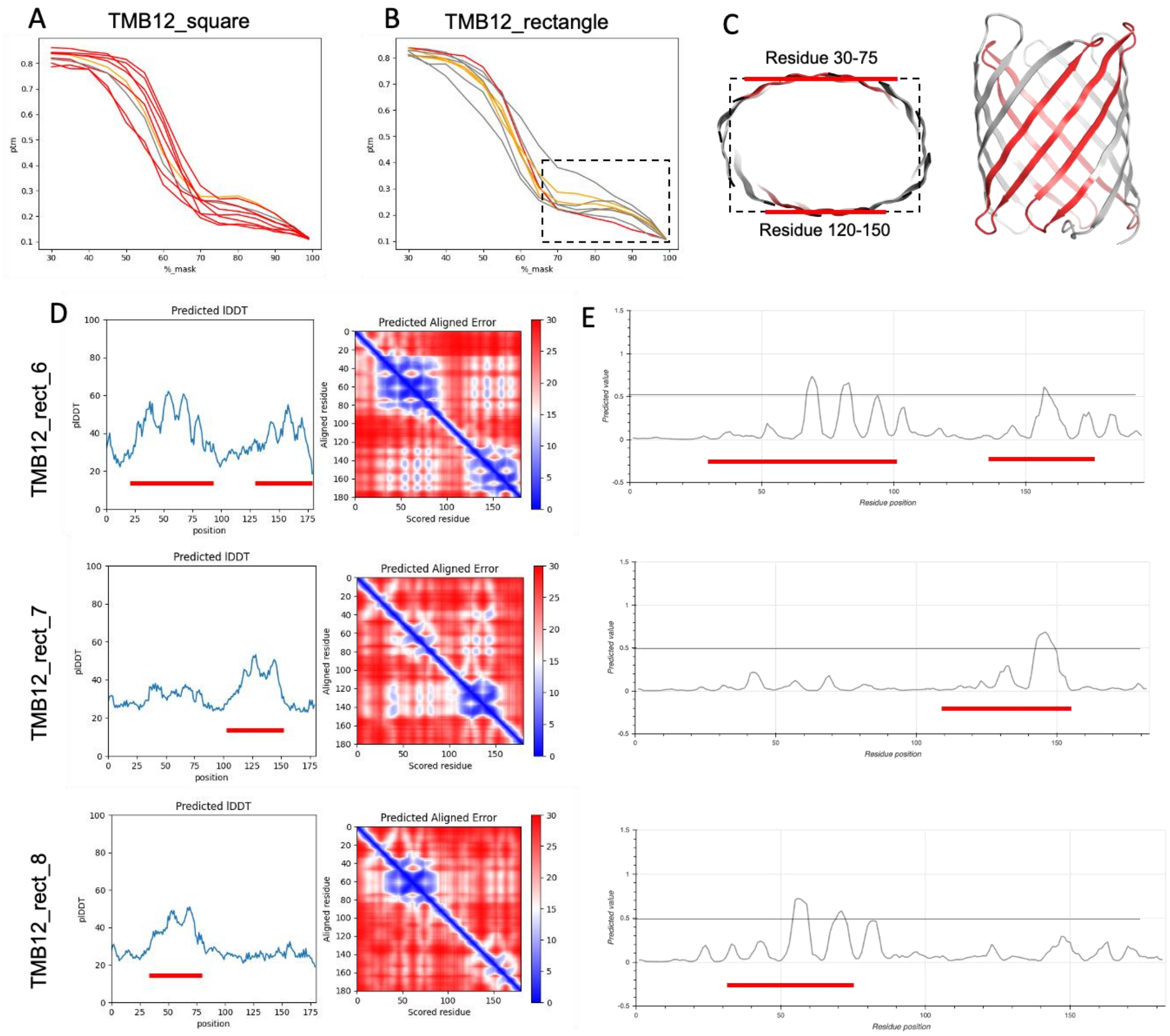

Figure S21: The square-shaped TMB12 designs (A) express at higher levels (red=strongly expressed; orange=weakly expressed; gray=no expression) than rectangle-shaped designs (B) and feature less residual secondary structure content at the end of ESM in silico-melting (*32*) simulations (highlighted by a dashed rectangle in B). Closer analysis of the ESM simulations show that the regions of melting-resistant secondary structure correspond to the long sides of the designed rectangular TMB12 structures (C, D) and co-localize with early-folding regions predicted with EFoldmine (*43*) (E).

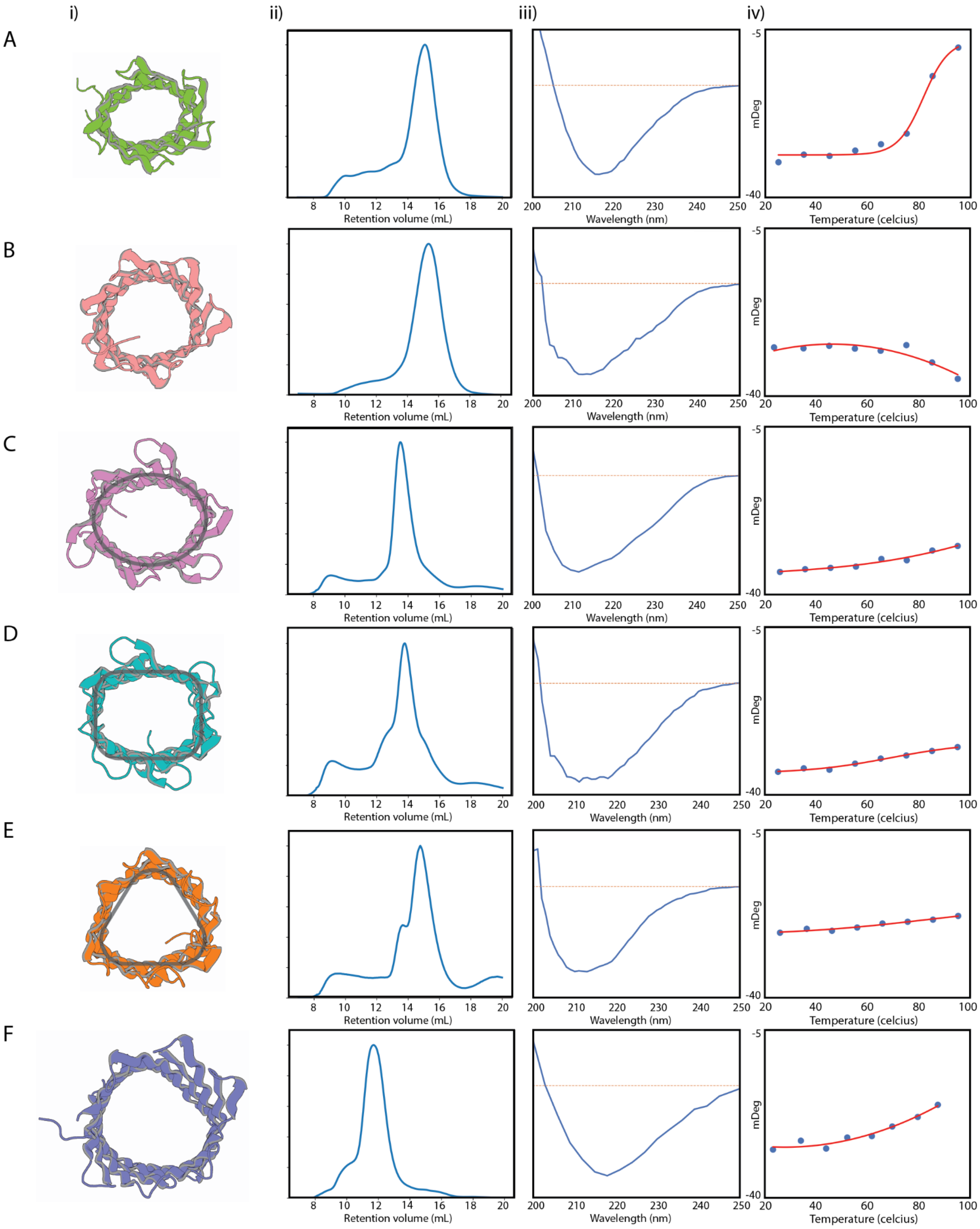

Figure S22: Characterisation of designed TMB pores. A. TMB10_165, B. TMB12_3, C. TMB12_oval_4, D. TMB12_rect_8, E. TMB12_tri_12, F. TMB14_8. i) Respective cartoons indicating top view of the designs. ii) Size Exclusion Chromatography plots for all designs carried out in a buffer containing 0.1% DPC detergent. iii) Corresponding Circular Dichroism (CD) plots in the near-UV range. iv) CD melt plots from 25 C to 95 C.

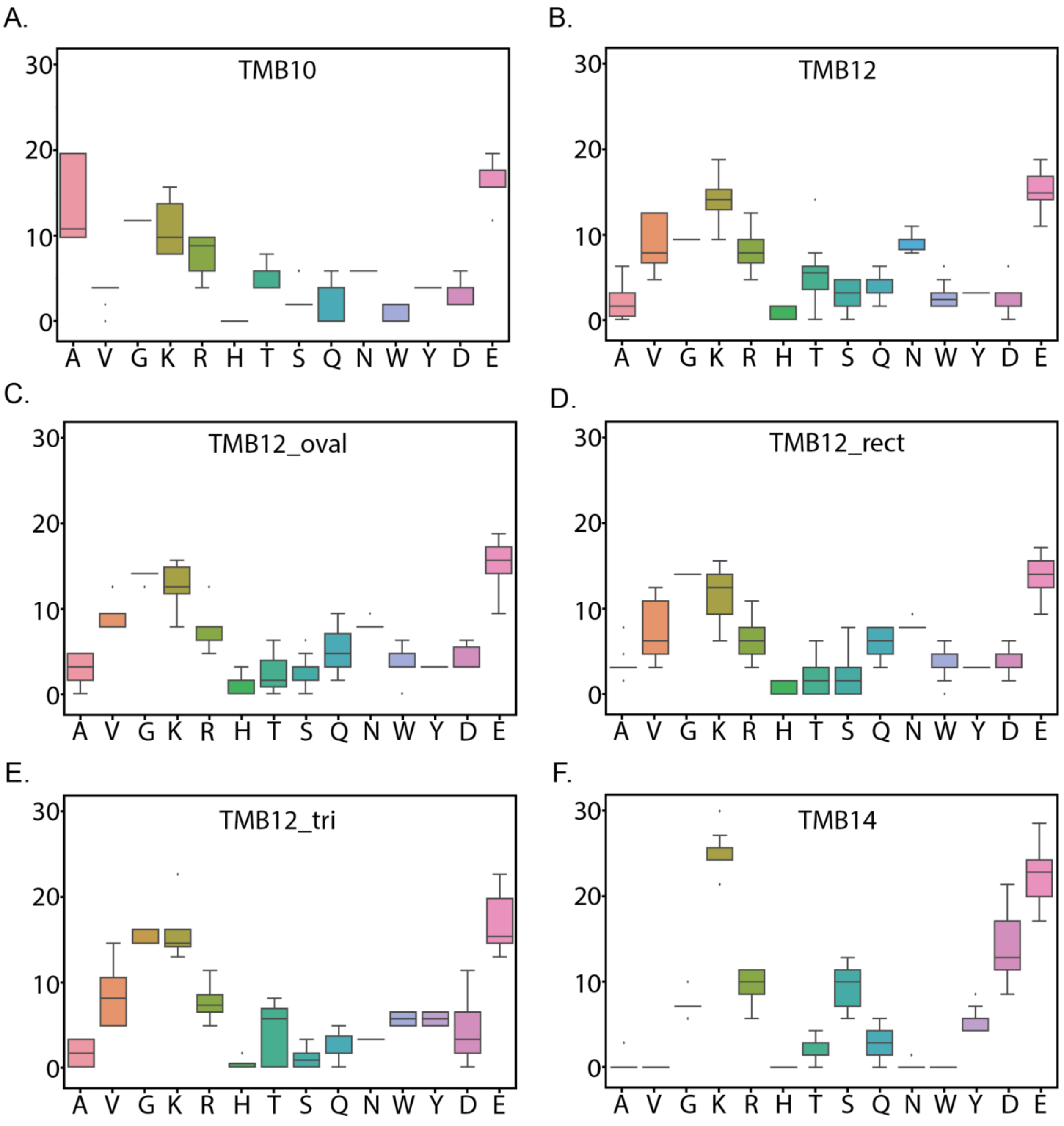

Figure S23: Pore lining amino acid compositions of the different types of designs. Y-axis indicates the percent fraction of the total number of a specific amino-acid within all pore-lining residues.

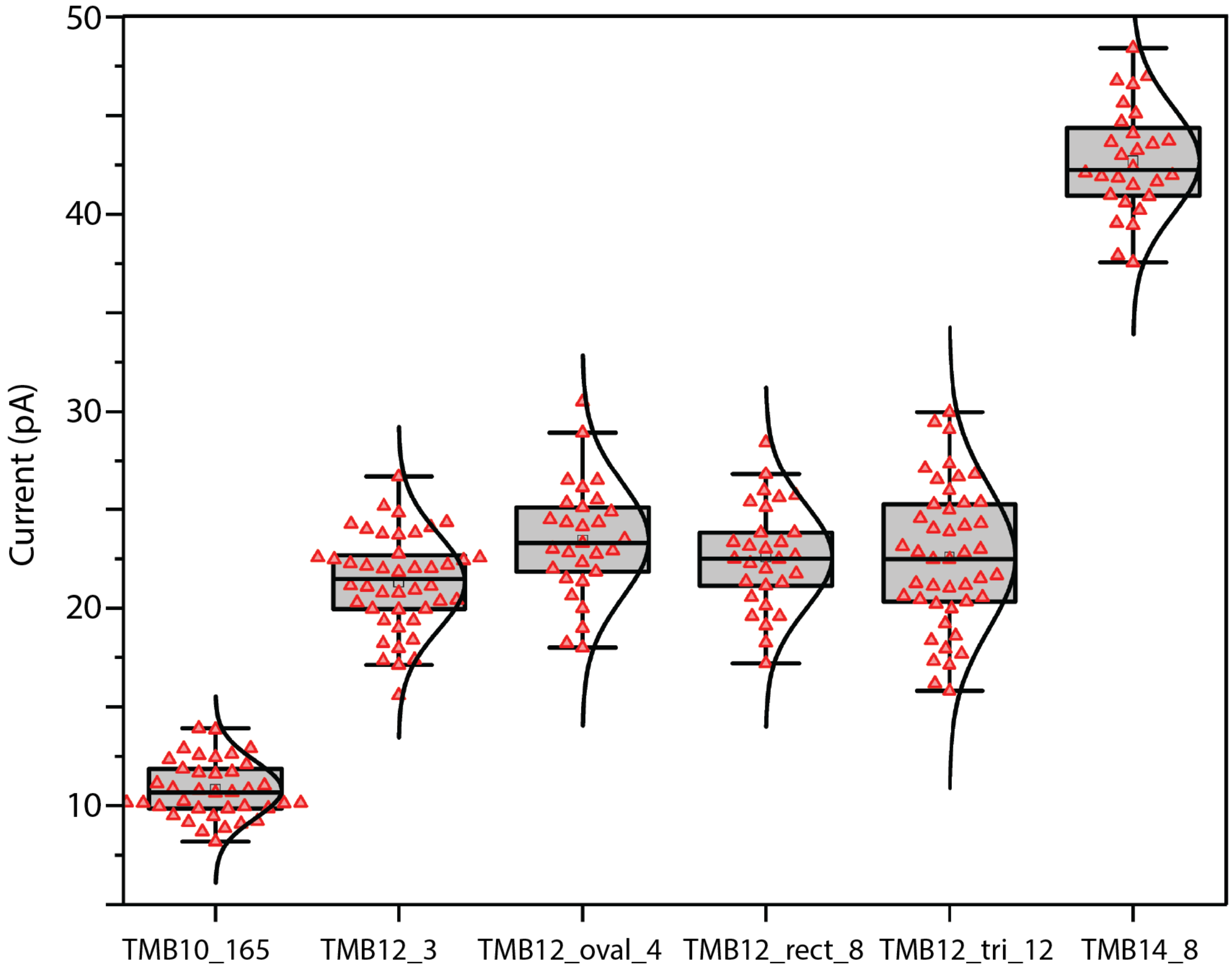

Figure S24: An all points box plot of the minimal step conductances observed across 3 independent recordings for each of the 6 designs shown in Figure 4. The voltage was kept constant at 100 mV throughout the recordings and across all current jumps. Both *cis* and *trans* solutions had 500 mM NaCl as the electrolyte.

References and Notes

1. M. Dal Peraro, F. G. van der Goot, Pore-forming toxins: Ancient, but never really out of fashion. *Nat. Rev. Microbiol.* **14**, 77–92 (2016). doi:10.1038/nrmicro.2015.3 Medline

2. D. Ranava, A. Caumont-Sarcos, C. Albenne, R. Ieva, Bacterial machineries for the assembly of membrane-embedded β-barrel proteins. *FEMS Microbiol. Lett.* **365**, fny087 (2018). doi:10.1093/femsle/fny087 Medline

3. K. A. Diederichs, S. K. Buchanan, I. Botos, Building Better Barrels - β-barrel Biogenesis and Insertion in Bacteria and Mitochondria. *J. Mol. Biol.* **433**, 166894 (2021). doi:10.1016/j.jmb.2021.166894 Medline

4. P. M. Day, K. Inoue, S. M. Theg, Chloroplast Outer Membrane β-Barrel Proteins Use Components of the General Import Apparatus. *Plant Cell* **31**, 1845–1855 (2019). doi:10.1105/tpc.19.00001 Medline

5. L. Harrington, L. T. Alexander, S. Knapp, H. Bayley, Single-Molecule Protein Phosphorylation and Dephosphorylation by Nanopore Enzymology. *ACS Nano* **13**, 633–641 (2019). doi:10.1021/acsnano.8b07697 Medline

6. M. Afshar Bakshloo, J. J. Kasianowicz, M. Pastoriza-Gallego, J. Mathé, R. Daniel, F. Piguet, A. Oukhaled, Nanopore-Based Protein Identification. *J. Am. Chem. Soc.* **144**, 2716–2725 (2022). doi:10.1021/jacs.1c11758 Medline

7. M. A. V. Fahie, B. Yang, C. M. Chisholm, M. Chen, Protein Analyte Sensing with an Outer Membrane Protein G (OmpG) Nanopore. *Methods Mol. Biol.* **2186**, 77–94 (2021). doi:10.1007/978-1-0716-0806-7_7 Medline

8. C. Cao, L. F. Krapp, A. Al Ouahabi, N. F. König, N. Cirauqui, A. Radenovic, J.-F. Lutz, M. D. Peraro, Aerolysin nanopores decode digital information stored in tailored macromolecular analytes. *Sci. Adv.* **6**, eabc2661 (2020). doi:10.1126/sciadv.abc2661 Medline

9. S. E. Van der Verren, N. Van Gerven, W. Jonckheere, R. Hambley, P. Singh, J. Kilgour, M. Jordan, E. J. Wallace, L. Jayasinghe, H. Remaut, A dual-constriction biological nanopore resolves homonucleotide sequences with high fidelity. *Nat. Biotechnol.* **38**, 1415–1420 (2020). doi:10.1038/s41587-020-0570-8 Medline

10. D. Deamer, M. Akeson, D. Branton, Three decades of nanopore sequencing. *Nat. Biotechnol.* **34**, 518–524 (2016). doi:10.1038/nbt.3423 Medline

11. C. Cao, N. Cirauqui, M. J. Marcaida, E. Buglakova, A. Duperrex, A. Radenovic, M. Dal Peraro, Single-molecule sensing of peptides and nucleic acids by engineered aerolysin nanopores. *Nat. Commun.* **10**, 4918 (2019). doi:10.1038/s41467-019-12690-9 Medline

12. K. Shimizu, B. Mijiddorj, M. Usami, I. Mizoguchi, S. Yoshida, S. Akayama, Y. Hamada, A. Ohyama, K. Usui, I. Kawamura, R. Kawano, De novo design of a nanopore for single-molecule detection that incorporates a β-hairpin peptide. *Nat. Nanotechnol.* **17**, 67–75 (2022). doi:10.1038/s41565-021-01008-w Medline

13. A. A. Vorobieva, P. White, B. Liang, J. E. Horne, A. K. Bera, C. M. Chow, S. Gerben, S. Marx, A. Kang, A. Q. Stiving, S. R. Harvey, D. C. Marx, G. N. Khan, K. G. Fleming, V.

H. Wysocki, D. J. Brockwell, L. K. Tamm, S. E. Radford, D. Baker, De novo design of transmembrane β barrels. *Science* **371**, eabc8182 (2021). doi:10.1126/science.abc8182 Medline

14. W. M. Liu, Shear numbers of protein beta-barrels: Definition refinements and statistics. *J. Mol. Biol.* **275**, 541–545 (1998). doi:10.1006/jmbi.1997.1501 Medline
15. A. G. Murzin, A. M. Lesk, C. Chothia, Principles determining the structure of β-sheet barrels in proteins. I. A theoretical analysis. *J. Mol. Biol.* **236**, 1369–1381 (1994). doi:10.1016/0022-2836(94)90064-7 Medline
16. M. W. Franklin, J. S. G. Slusky, Tight Turns of Outer Membrane Proteins: An Analysis of Sequence, Structure, and Hydrogen Bonding. *J. Mol. Biol.* **430** (18 Pt B), 3251–3265 (2018). doi:10.1016/j.jmb.2018.06.013 Medline
17. B. Pham, C. M. Chisholm, J. Foster, E. Friis, M. A. Fahie, M. Chen, A pH-independent quiet OmpG pore with enhanced electrostatic repulsion among the extracellular loops. *BBA* **1863**, 183485 (2021). doi:10.1016/j.bbamem.2020.183485 Medline
18. M. Chen, S. Khalid, M. S. P. Sansom, H. Bayley, Outer membrane protein G: Engineering a quiet pore for biosensing. *Proc. Natl. Acad. Sci. U.S.A.* **105**, 6272–6277 (2008). doi:10.1073/pnas.0711561105 Medline
19. N. Koga, R. Tatsumi-Koga, G. Liu, R. Xiao, T. B. Acton, G. T. Montelione, D. Baker, Principles for designing ideal protein structures. *Nature* **491**, 222–227 (2012). doi:10.1038/nature11600 Medline
20. J. Dou, A. A. Vorobieva, W. Sheffler, L. A. Doyle, H. Park, M. J. Bick, B. Mao, G. W. Foight, M. Y. Lee, L. A. Gagnon, L. Carter, B. Sankaran, S. Ovchinnikov, E. Marcos, P.-S. Huang, J. C. Vaughan, B. L. Stoddard, D. Baker, De novo design of a fluorescence-activating β-barrel. *Nature* **561**, 485–491 (2018). doi:10.1038/s41586-018-0509-0 Medline
21. F. R. Salemme, Conformational and geometrical properties of beta-sheets in proteins. III. Isotropically stressed configurations. *J. Mol. Biol.* **146**, 143–156 (1981). doi:10.1016/0022-2836(81)90370-3 Medline
22. T. Surrey, F. Jähnig, Kinetics of folding and membrane insertion of a β-barrel membrane protein. *J. Biol. Chem.* **270**, 28199–28203 (1995). doi:10.1074/jbc.270.47.28199 Medline
23. E. J. Danoff, K. G. Fleming, Novel Kinetic Intermediates Populated along the Folding Pathway of the Transmembrane β-Barrel OmpA. *Biochemistry* **56**, 47–60 (2017). doi:10.1021/acs.biochem.6b00809 Medline
24. E. Di Silvio, M. Brunori, S. Gianni, Frustration Sculpts the Early Stages of Protein Folding. *Angew. Chem. Int. Ed.* **54**, 10867–10869 (2015). doi:10.1002/anie.201504835 Medline
25. M. Michalik, M. Orwick-Rydmark, M. Habeck, V. Alva, T. Arnold, D. Linke, An evolutionarily conserved glycine-tyrosine motif forms a folding core in outer membrane proteins. *PLOS ONE* **12**, e0182016 (2017). doi:10.1371/journal.pone.0182016 Medline
26. D. L. Leyton, M. D. Johnson, R. Thapa, G. H. M. Huysmans, R. A. Dunstan, N. Celik, H.-H. Shen, D. Loo, M. J. Belousoff, A. W. Purcell, I. R. Henderson, T. Beddoe, J. Rossjohn, L. L. Martin, R. A. Strugnell, T. Lithgow, A mortise-tenon joint in the transmembrane

domain modulates autotransporter assembly into bacterial outer membranes. *Nat. Commun.* **5**, 4239 (2014). doi:10.1038/ncomms5239 Medline

27. R. F. Alford, A. Leaver-Fay, J. R. Jeliazkov, M. J. O'Meara, F. P. DiMaio, H. Park, M. V. Shapovalov, P. D. Renfrew, V. K. Mulligan, K. Kappel, J. W. Labonte, M. S. Pacella, R. Bonneau, P. Bradley, R. L. Dunbrack Jr., R. Das, D. Baker, B. Kuhlman, T. Kortemme, J. J. Gray, The Rosetta All-Atom Energy Function for Macromolecular Modeling and Design. *J. Chem. Theory Comput.* **13**, 3031–3048 (2017). doi:10.1021/acs.jctc.7b00125 Medline

28. Z. Wang, F. Zhao, J. Peng, J. Xu, Protein 8-class secondary structure prediction using conditional neural fields. *Proteomics* **11**, 3786–3792 (2011). doi:10.1002/pmic.201100196 Medline

29. A.-M. Fernandez-Escamilla, F. Rousseau, J. Schymkowitz, L. Serrano, Prediction of sequence-dependent and mutational effects on the aggregation of peptides and proteins. *Nat. Biotechnol.* **22**, 1302–1306 (2004). doi:10.1038/nbt1012 Medline

30. J. Jumper, R. Evans, A. Pritzel, T. Green, M. Figurnov, O. Ronneberger, K. Tunyasuvunakool, R. Bates, A. Žídek, A. Potapenko, A. Bridgland, C. Meyer, S. A. A. Kohl, A. J. Ballard, A. Cowie, B. Romera-Paredes, S. Nikolov, R. Jain, J. Adler, T. Back, S. Petersen, D. Reiman, E. Clancy, M. Zielinski, M. Steinegger, M. Pacholska, T. Berghammer, S. Bodenstein, D. Silver, O. Vinyals, A. W. Senior, K. Kavukcuoglu, P. Kohli, D. Hassabis, Highly accurate protein structure prediction with AlphaFold. *Nature* **596**, 583–589 (2021). doi:10.1038/s41586-021-03819-2 Medline

31. M. Mirdita, K. Schütze, Y. Moriwaki, L. Heo, S. Ovchinnikov, M. Steinegger, ColabFold: Making protein folding accessible to all. *Nat. Methods* **19**, 679–682 (2022). doi:10.1038/s41592-022-01488-1 Medline

32. A. M. Hermosilla, C. Berner, S. Ovchinnikov, A. A. Vorobieva, Validation of de novo designed water-soluble and transmembrane proteins by in silico folding and melting. bioRxiv 2023.06.06.543955 [Preprint] (2023); doi:10.1101/2023.06.06.543955v1.

33. A. Konovalova, D. E. Kahne, T. J. Silhavy, Outer Membrane Biogenesis. *Annu. Rev. Microbiol.* **71**, 539–556 (2017). doi:10.1146/annurev-micro-090816-093754 Medline

34. B. Schiffrin, D. J. Brockwell, S. E. Radford, Outer membrane protein folding from an energy landscape perspective. *BMC Biol.* **15**, 123 (2017). doi:10.1186/s12915-017-0464-5 Medline

35. N. K. Burgess, T. P. Dao, A. M. Stanley, K. G. Fleming, Beta-barrel proteins that reside in the Escherichia coli outer membrane in vivo demonstrate varied folding behavior in vitro. *J. Biol. Chem.* **283**, 26748–26758 (2008). doi:10.1074/jbc.M802754200 Medline

36. H. Hong, L. K. Tamm, Elastic coupling of integral membrane protein stability to lipid bilayer forces. *Proc. Natl. Acad. Sci. U.S.A.* **101**, 4065–4070 (2004). doi:10.1073/pnas.0400358101 Medline

37. C. P. Moon, N. R. Zaccai, P. J. Fleming, D. Gessmann, K. G. Fleming, Membrane protein thermodynamic stability may serve as the energy sink for sorting in the periplasm. *Proc. Natl. Acad. Sci. U.S.A.* **110**, 4285–4290 (2013). doi:10.1073/pnas.1212527110 Medline

38. C. P. Moon, K. G. Fleming, Side-chain hydrophobicity scale derived from transmembrane protein folding into lipid bilayers. *Proc. Natl. Acad. Sci. U.S.A.* **108**, 10174–10177 (2011). doi:10.1073/pnas.1103979108 Medline

39. H. Hong, S. Park, R. H. F. Jiménez, D. Rinehart, L. K. Tamm, Role of aromatic side chains in the folding and thermodynamic stability of integral membrane proteins. *J. Am. Chem. Soc.* **129**, 8320–8327 (2007). doi:10.1021/ja068849o Medline

40. J. K. Leman, B. D. Weitzner, S. M. Lewis, J. Adolf-Bryfogle, N. Alam, R. F. Alford, M. Aprahamian, D. Baker, K. A. Barlow, P. Barth, B. Basanta, B. J. Bender, K. Blacklock, J. Bonet, S. E. Boyken, P. Bradley, C. Bystroff, P. Conway, S. Cooper, B. E. Correia, B. Coventry, R. Das, R. M. De Jong, F. DiMaio, L. Dsilva, R. Dunbrack, A. S. Ford, B. Frenz, D. Y. Fu, C. Geniesse, L. Goldschmidt, R. Gowthaman, J. J. Gray, D. Gront, S. Guffy, S. Horowitz, P.-S. Huang, T. Huber, T. M. Jacobs, J. R. Jeliazkov, D. K. Johnson, K. Kappel, J. Karanicolas, H. Khakzad, K. R. Khar, S. D. Khare, F. Khatib, A. Khramushin, I. C. King, R. Kleffner, B. Koepnick, T. Kortemme, G. Kuenze, B. Kuhlman, D. Kuroda, J. W. Labonte, J. K. Lai, G. Lapidoth, A. Leaver-Fay, S. Lindert, T. Linsky, N. London, J. H. Lubin, S. Lyskov, J. Maguire, L. Malmström, E. Marcos, O. Marcu, N. A. Marze, J. Meiler, R. Moretti, V. K. Mulligan, S. Nerli, C. Norn, S. Ó'Conchúir, N. Ollikainen, S. Ovchinnikov, M. S. Pacella, X. Pan, H. Park, R. E. Pavlovicz, M. Pethe, B. G. Pierce, K. B. Pilla, B. Raveh, P. D. Renfrew, S. S. R. Burman, A. Rubenstein, M. F. Sauer, A. Scheck, W. Schief, O. Schueler-Furman, Y. Sedan, A. M. Sevy, N. G. Sgourakis, L. Shi, J. B. Siegel, D.-A. Silva, S. Smith, Y. Song, A. Stein, M. Szegedy, F. D. Teets, S. B. Thyme, R. Y.-R. Wang, A. Watkins, L. Zimmerman, R. Bonneau, Macromolecular modeling and design in Rosetta: Recent methods and frameworks. *Nat. Methods* **17**, 665–680 (2020). doi:10.1038/s41592-020-0848-2 Medline

41. M. Pellegrini-Calace, T. Maiwald, J. M. Thornton, PoreWalker: A novel tool for the identification and characterization of channels in transmembrane proteins from their three-dimensional structure. *PLOS Comput. Biol.* **5**, e1000440 (2009). doi:10.1371/journal.pcbi.1000440 Medline

42. L. Pravda, D. Sehnal, D. Toušek, V. Navrátilová, V. Bazgier, K. Berka, R. Svobodová Vareková, J. Koca, M. Otyepka, MOLEonline: A web-based tool for analyzing channels, tunnels and pores (2018 update). *Nucleic Acids Res.* **46**, W368–W373 (2018). doi:10.1093/nar/gky309 Medline

43. D. Raimondi, G. Orlando, R. Pancsa, T. Khan, W. F. Vranken, Exploring the Sequence-based Prediction of Folding Initiation Sites in Proteins. *Sci. Rep.* **7**, 8826 (2017). doi:10.1038/s41598-017-08366-3 Medline

44. S. W. Kowalczyk, A. Y. Grosberg, Y. Rabin, C. Dekker, Modeling the conductance and DNA blockade of solid-state nanopores. *Nanotechnology* **22**, 315101 (2011). doi:10.1088/0957-4484/22/31/315101 Medline

45. R. R. Sanganna Gari, P. Seelheim, B. Liang, L. K. Tamm, G. Quiet Outer Membrane Protein, Quiet Outer Membrane Protein G (OmpG) Nanopore for Biosensing. *ACS Sens.* **4**, 1230–1235 (2019). doi:10.1021/acssensors.8b01645 Medline

46. C. Xu, P. Lu, T. M. Gamal El-Din, X. Y. Pei, M. C. Johnson, A. Uyeda, M. J. Bick, Q. Xu, D. Jiang, H. Bai, G. Reggiano, Y. Hsia, T. J. Brunette, J. Dou, D. Ma, E. M. Lynch, S. E.

Boyken, P.-S. Huang, L. Stewart, F. DiMaio, J. M. Kollman, B. F. Luisi, T. Matsuura, W. A. Catterall, D. Baker, Computational design of transmembrane pores. *Nature* **585**, 129–134 (2020). doi:10.1038/s41586-020-2646-5 Medline

47. H. T. Kratochvil, L. C. Watkins, M. Mravic, J. L. Thomaston, J. M. Nicoludis, N. H. Somberg, L. Liu, M. Hong, G. A. Voth, W. F. DeGrado, Transient water wires mediate selective proton transport in designed channel proteins. *Nat. Chem.* **15**, 1012–1021 (2023). doi:10.1038/s41557-023-01210-4 Medline

48. K. R. Mahendran, A. Niitsu, L. Kong, A. R. Thomson, R. B. Sessions, D. N. Woolfson, H. Bayley, A monodisperse transmembrane α-helical peptide barrel. *Nat. Chem.* **9**, 411–419 (2017). doi:10.1038/nchem.2647 Medline

49. A. J. Scott, A. Niitsu, H. T. Kratochvil, E. J. M. Lang, J. T. Sengel, W. M. Dawson, K. R. Mahendran, M. Mravic, A. R. Thomson, R. L. Brady, L. Liu, A. J. Mulholland, H. Bayley, W. F. DeGrado, M. I. Wallace, D. N. Woolfson, Constructing ion channels from water-soluble α-helical barrels. *Nat. Chem.* **13**, 643–650 (2021). doi:10.1038/s41557-021-00688-0 Medline

50. M. A. Fahie, J. Candido, G. Andree, M. Chen, Tuning Protein Discrimination Through Altering the Sampling Interface Formed between the Analyte and the OmpG Nanopore. *ACS Sens.* **6**, 1286–1294 (2021). doi:10.1021/acssensors.0c02580 Medline

51. J. C. Foster, B. Pham, R. Pham, M. Kim, M. D. Moore, M. Chen, An Engineered OmpG Nanopore with Displayed Peptide Motifs for Single-Molecule Multiplex Protein Detection. *Angew. Chem. Int. Ed.* **62**, e202214566 (2023). doi:10.1002/anie.202214566 Medline

52. H.-J. Hwang, J.-S. Kim, J. Lee, J. S. Min, K.-B. Jeong, E. Kim, M.-K. Lee, S.-W. Chi, Single-Molecule Sensing of an Anticancer Therapeutic Protein-Protein Interaction Using the Chemically Modified OmpG Nanopore. *Anal. Chem.* **94**, 7449–7454 (2022). doi:10.1021/acs.analchem.1c04840 Medline

53. M. A. Fahie, B. Yang, M. Mullis, M. A. Holden, M. Chen, Selective Detection of Protein Homologues in Serum Using an OmpG Nanopore. *Anal. Chem.* **87**, 11143–11149 (2015). doi:10.1021/acs.analchem.5b03350 Medline

54. M. A. Fahie, M. Chen, Electrostatic Interactions between OmpG Nanopore and Analyte Protein Surface Can Distinguish between Glycosylated Isoforms. *J. Phys. Chem. B* **119**, 10198–10206 (2015). doi:10.1021/acs.jpcb.5b06435 Medline

55. L. An, M. Said, L. Tran, S. Majumder, I. Goreshnik, G. R. Lee, D. Juergens, J. Dauparas, I. Anishchenko, B. Coventry, A. K. Bera, A. Kang, P. M. Levine, V. Alvarez, A. Pillai, C. Norn, D. Feldman, D. Zorine, D. R. Hicks, X. Li, M. G. Sanchez, D. K. Vafeados, P. J. Salveson, A. A. Vorobieva, D. Baker, De novo design of diverse small molecule binders and sensors using Shape Complementary Pseudocycles. *Science* **385**, 276–282 (2024). doi:10.1126/science.adn3780.

56. J. A. Peruzzi, J. Steinkühler, T. Q. Vu, T. F. Gunnels, P. Lu, D. Baker, N. P. Kamat, Hydrophobic mismatch drives self-organization of designer proteins into synthetic membranes. *Nature* **15**, 3162 (2024). doi:10.1038/s41467-024-47163-1 Medline.

57. A. Sauciuc, B. Morozzo Della Rocca, M. J. Tadema, M. Chinappi, G. Maglia, Translocation of linearized full-length proteins through an engineered nanopore under opposing electrophoretic force. *Nat. Biotechnol.* (2023). doi:10.1038/s41587-023-01954-x Medline

58. J. Liu, A. Aksimentiev, Molecular Determinants of Current Blockade Produced by Peptide Transport Through a Nanopore. *ACS Nanosci. Au* **4**, 21–29 (2023). doi:10.1021/acsnanoscienceau.3c00046 Medline

59. A. Vorobieva, demo_TMB_design: demo_nanopore_design. Version nanopore_design_pub, Zenodo (2024); https://zenodo.org/records/10935638

60. S. Majumder, MATLAB analysis scripts for denovo Nanopore ion conductance data. Version v1, Zenodo (2024); https://zenodo.org/records/10939541

61. W. Kabsch, XDS. *Acta Crystallogr. D.* **66**, 125–132 (2010). doi:10.1107/S0907444909047337 Medline

62. M. D. Winn, C. C. Ballard, K. D. Cowtan, E. J. Dodson, P. Emsley, P. R. Evans, R. M. Keegan, E. B. Krissinel, A. G. W. Leslie, A. McCoy, S. J. McNicholas, G. N. Murshudov, N. S. Pannu, E. A. Potterton, H. R. Powell, R. J. Read, A. Vagin, K. S. Wilson, Overview of the CCP4 suite and current developments. *Acta Crystallogr. D.* **67**, 235–242 (2011). doi:10.1107/S0907444910045749 Medline

63. A. J. McCoy, R. W. Grosse-Kunstleve, P. D. Adams, M. D. Winn, L. C. Storoni, R. J. Read, Phaser crystallographic software. *J. Appl. Cryst.* **40**, 658–674 (2007). doi:10.1107/S0021889807021206 Medline

64. P. D. Adams, P. V. Afonine, G. Bunkóczi, V. B. Chen, I. W. Davis, N. Echols, J. J. Headd, L.-W. Hung, G. J. Kapral, R. W. Grosse-Kunstleve, A. J. McCoy, N. W. Moriarty, R. Oeffner, R. J. Read, D. C. Richardson, J. S. Richardson, T. C. Terwilliger, P. H. Zwart, PHENIX: A comprehensive Python-based system for macromolecular structure solution. *Acta Crystallogr. D.* **66**, 213–221 (2010). doi:10.1107/S0907444909052925 Medline

65. P. Emsley, K. Cowtan, Coot: Model-building tools for molecular graphics. *Acta Crystallogr. D.* **60**, 2126–2132 (2004). doi:10.1107/S0907444904019158 Medline

66. C. J. Williams, J. J. Headd, N. W. Moriarty, M. G. Prisant, L. L. Videau, L. N. Deis, V. Verma, D. A. Keedy, B. J. Hintze, V. B. Chen, S. Jain, S. M. Lewis, W. B. Arendall 3rd, J. Snoeyink, P. D. Adams, S. C. Lovell, J. S. Richardson, D. C. Richardson, MolProbity: More and better reference data for improved all-atom structure validation. *Protein Sci.* **27**, 293–315 (2018). doi:10.1002/pro.3330 Medline

67. A. Favier, B. Brutscher, NMRlib: User-friendly pulse sequence tools for Bruker NMR spectrometers. *J. Biomol. NMR* **73**, 199–211 (2019). doi:10.1007/s10858-019-00249-1 Medline

68. Y. Xia, K. Sze, G. Zhu, Transverse relaxation optimized 3D and 4D 15n/15N separated NOESY experiments of 15N labeled proteins. *J. Biomol. NMR* **18**, 261–268 (2000). doi:10.1023/A:1026590201757 Medline

69. V. Tugarinov, R. Sprangers, L. E. Kay, Line narrowing in methyl-TROSY using zero-quantum 1H-13C NMR spectroscopy. *J. Am. Chem. Soc.* **126**, 4921–4925 (2004). doi:10.1021/ja039732s Medline

70. P. Güntert, L. Buchner, Combined automated NOE assignment and structure calculation with CYANA. *J. Biomol. NMR* **62**, 453–471 (2015). doi:10.1007/s10858-015-9924-9 Medline

71. F. Delaglio, S. Grzesiek, G. W. Vuister, G. Zhu, J. Pfeifer, A. Bax, NMRPipe: A multidimensional spectral processing system based on UNIX pipes. *J. Biomol. NMR* **6**, 277–293 (1995). doi:10.1007/BF00197809 Medline

72. S. P. Skinner, R. H. Fogh, W. Boucher, T. J. Ragan, L. G. Mureddu, G. W. Vuister, CcpNmr AnalysisAssign: A flexible platform for integrated NMR analysis. *J. Biomol. NMR* **66**, 111–124 (2016). doi:10.1007/s10858-016-0060-y Medline

73. Y. Shen, A. Bax, "Protein Structural Information Derived from NMR Chemical Shift with the Neural Network Program TALOS-N", in Methods in Molecular Biology. (Springer, 2015); vol 1260, pp. 17–32.

74. "Determination and analysis of urea and guanidine hydrochloride denaturation curves" in *Methods in Enzymology*, vol. 131 (Academic Press, 1986), pp. 266–280.

75. A. A. Vorobieva, Rationale in Custom Design of Transmembrane β-Barrel Pores. *Methods Mol. Biol.* **2778**, 345–366 (2024). doi:10.1007/978-1-0716-3734-0_21 Medline

76. S. E. Boyken, Z. Chen, B. Groves, R. A. Langan, G. Oberdorfer, A. Ford, J. M. Gilmore, C. Xu, F. DiMaio, J. H. Pereira, B. Sankaran, G. Seelig, P. H. Zwart, D. Baker, De novo design of protein homo-oligomers with modular hydrogen-bond network-mediated specificity. *Science* **352**, 680–687 (2016). doi:10.1126/science.aad8865 Medline

77. Y. Zhang, J. Skolnick, TM-align: A protein structure alignment algorithm based on the TM-score. *Nucleic Acids Res.* **33**, 2302–2309 (2005). doi:10.1093/nar/gki524 Medline
